# AKT and MAPK signaling pathways in hippocampus reveals the pathogenesis of depression in four stress-induced models

**DOI:** 10.1038/s41398-023-02486-3

**Published:** 2023-06-12

**Authors:** Xuemei Li, Teng Teng, Wei Yan, Li Fan, Xueer Liu, Gerard Clarke, Dan Zhu, Yuanliang Jiang, Yajie Xiang, Ying Yu, Yuqing Zhang, Bangmin Yin, Lin Lu, Xinyu Zhou, Peng Xie

**Affiliations:** 1 Department of Psychiatry, The First Affiliated Hospital of Chongqing Medical University, Chongqing, China; 2 NHC Key Laboratory of Diagnosis and Treatment on Brain Functional Diseases, The First Affiliated Hospital of Chongqing Medical University, Chongqing, China; 3 Peking University Sixth Hospital, Peking University Institute of Mental Health, NHC Key Laboratory of Mental Health (Peking University), National Clinical Research Center for Mental Disorders (Peking University Sixth Hospital), Beijing, China; 4 Department of Neurology, The First Affiliated Hospital of Chongqing Medical University, Chongqing, China; 5 Department of Psychiatry and Neurobehavioural Science, University College Cork, Cork, Ireland; 6 APC Microbiome Ireland, University College Cork, Cork, Ireland; 7 Department of Neurology, The Second Affiliated Hospital of Chongqing Medical University, Chongqing, China

**Keywords:** Molecular neuroscience, Depression

## Abstract

Major depressive disorder (MDD) is a highly heterogeneous psychiatric disorder. The pathogenesis of MDD remained unclear, and it may be associated with exposure to different stressors. Most previous studies have focused on molecular changes in a single stress-induced depression model, which limited the identification of the pathogenesis of MDD. The depressive-like behaviors were induced by four well-validated stress models in rats, including chronic unpredictable mild stress, learned helplessness stress, chronic restraint stress and social defeat stress. We applied proteomic and metabolomic to investigate molecular changes in the hippocampus of those four models and revealed 529 proteins and 98 metabolites. Ingenuity Pathways Analysis (IPA) and Kyoto Encyclopedia of Genes and Genomes (KEGG) analysis identified differentially regulated canonical pathways, and then we presented a schematic model that simulates AKT and MAPK signaling pathways network and their interactions and revealed the cascade reactions. Further, the western blot confirmed that p-AKT, p-ERK12, GluA1, p-MEK1, p-MEK2, p-P38, Syn1, and TrkB, which were changed in at least one depression model. Importantly, p-AKT, p-ERK12, p-MEK1 and p-P38 were identified as common alterations in four depression models. The molecular level changes caused by different stressors may be dramatically different, and even opposite, between four depression models. However, the different molecular alterations converge on a common AKT and MAPK molecular pathway. Further studies of these pathways could contribute to a better understanding of the pathogenesis of depression, with the ultimate goal of helping to develop or select more effective treatment strategies for MDD.

## Introduction

Major depressive disorder (MDD) is the leading cause of disability globally, with over 300 million people suffering from depression worldwide [1], and it is projected to be the second leading cause of disease and disability globally by 2030 [2]. The complexity and heterogeneity of depression make it difficult to identify a single underlying abnormality and suggests that there are multiple causes of depression [3]. Although the underlying mechanism of MDD remains elusive, stress has been recognized as a determinant risk factor of depression [4, 5]. In addition, stress is a heterogeneous phenomenon, and different types of recent life events and stressors may exert their effects via different neurobiological pathways and mechanisms, before leading to the emergence of depression [6]. Thus, it is importance to investigate the common and/or diverging pathogenesis of depression caused by different types of stress.

Stress-induced depression models have been widely used and developed as an important tool for exploring the complex pathogenesis of MDD for over 30 years [7, 8]. There are several stress-induced depression models to mimic the different stressors encountered in human daily life [9]. For example, the chronic unpredictable mild stress (CUMS) rodent model mimics the variable and unpredictable physical and mental irritations encountered in human daily life and reflect some of the core symptoms in depressed humans (e.g., anhedonia, anxiety and despair) [10], and the chronic restraint stress (CRS) model simulates deprivation of freedom [11]. The social defeat stress (SD) model mimics the pathogenesis of depression at a social level and explores the biological basis of stress resilience behavior that mimics characteristics of MDD (termed ‘susceptible’), while the remainder do not (termed ‘resilient’) [12]. The SD model thus recapitulates the differences in stress responses observed in humans [13]. The learned helplessness stress (LH) model serves as a general protocol where exposure to inescapable stress subsequently affects escape responding or ability to cope, presumably by inducing a state of “helplessness” [14]. More importantly, the results of different stress-induced depression models provide important insight into the heterogeneous findings.

The hippocampus is regarded as the key stress-responsive brain region involved in memory, learning and mood regulation, and plays an important role in the pathogenesis of depression [15]. Hippocampal structural volume reduction is related to stress and depression [16, 17], and hippocampal neurogenesis can buffer stress responses and depressive behavior [18]. However, different stressors can cause heterogeneous, even diametrically opposed, stress responses in the hippocampus [19], such as the expression of plasticity-related proteins [20]. However, the role of different stressors in mediating the pathogenesis of depression in hippocampus remains unclear.

Recent technological advances and efforts in scientific discovery have acquired an in-depth understanding of the molecular mechanisms of depression. Due to the expansion of ‘omics’ technologies, hundreds of putative molecular proteins and metabolites have been discovered whose presence (or altered levels—up or down regulation) could indicate depression [21]. Meanwhile, new approaches based on omics data integration are expected to play a key role in identifying and qualifying new mechanisms, which is why the focus of data analysis approaches has shifted from single-omics to multi-omics data integration [22, 23]. Overall, multi-omics data integration allows the joint analysis of multiple omics data types to provide a global view of the biological system and offers insights into the nature of the interactions between the different dataset layers [24].

However, most studies have typically focused on a single stress-induced depression model, and combination of different models can eventually decrease the etiological heterogeneity, and come closer to the clinical situation than a single, or even highly sophisticated model [25]. Thus, the combined multi-omics analysis and experimental verification to investigate the pathogenesis of multiple stress-induced models of depression may lay the foundation for further research.

## Materials and methods

A flowchart of the molecular profiling methods was shown in Supplementary Fig. S1.

### Animals

**Fig. 1:**
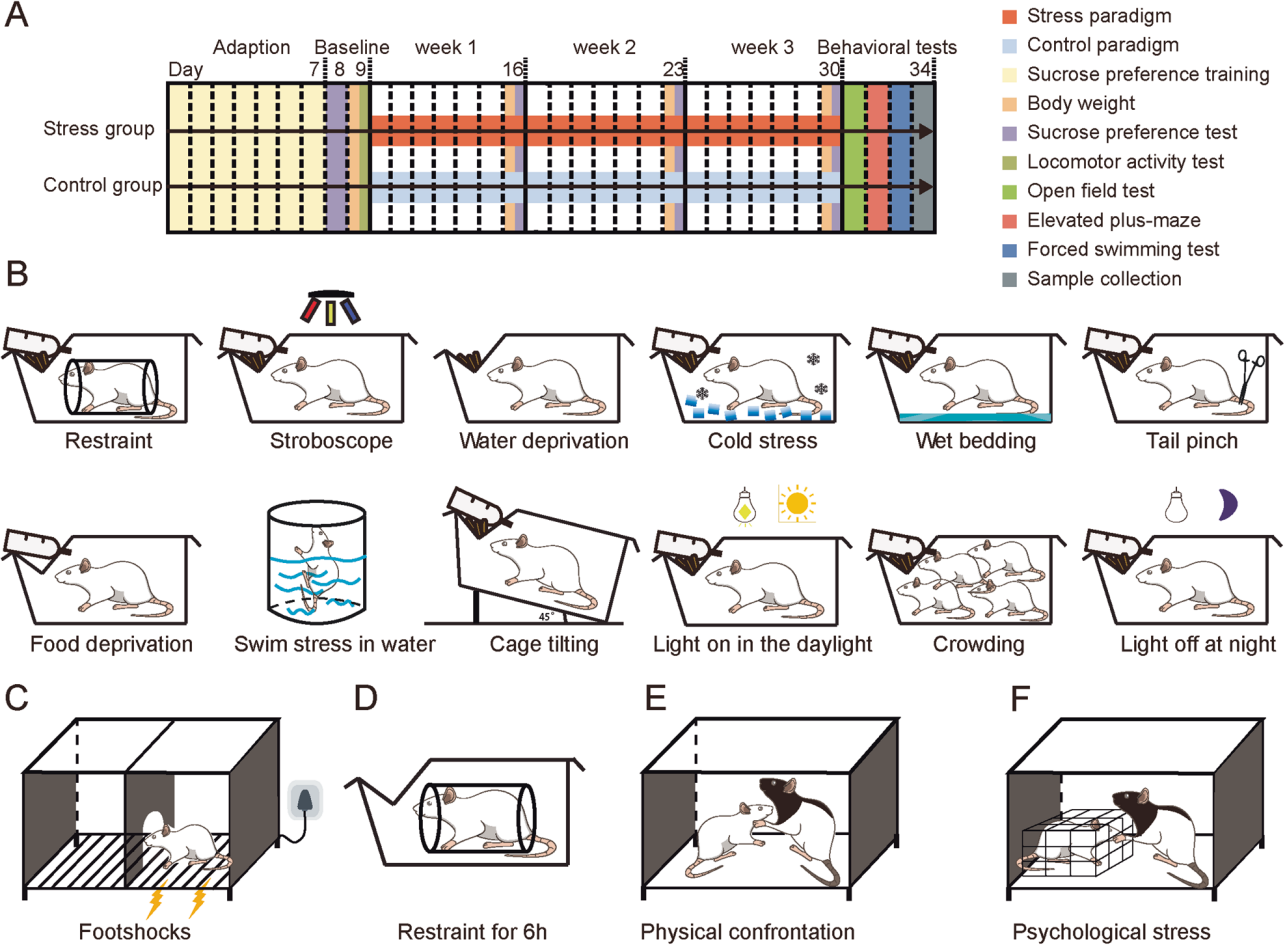
Procedure for the four depression models protocol. **A** The timeline of the stress and control groups regime and behavioral assessments. **B** The stimulus diagram of the CUMS model. **C** LH model. **D** CRS model. **E**, **F** SD model. CON, control group.

The animals were used as we published previously study [26]. One hundred and forty male Sprague-Dawley rats with an initial body weight of 200–300 g (8 weeks old) were obtained from animal facilities at Chongqing Medical University (Chongqing, China). All rats were fed in a single cage, and a 12-hour light/12-hour dark day-night regime (lights on at 19:00) with a constant temperature of 22 ± 1 °C and a relative humidity of 55 ± 5%. Food and water were abundant and freely available except under food and water deprivation. The experiments began after seven days of habituation to environmental conditions. The screening process based on locomotor activity test (LAT) and sucrose preference test (SPT). Then, the screened rats were randomly assigned to the experiment or control group. The schedule of the experimental program was shown in Fig. 1A. Animals were maintained in accordance with the guidelines of the National Institutes of Health [27] and approved by the Ethics Committee of Chongqing Medical University.

### Stress-induced depression models in rats

The paradigm of chronic unpredictable mild stress (CUMS), chronic restraint stress (CRS), social defeat (SD) and learned helplessness (LH) were reported in our previous studies [26, 28], and detailed in the Supplementary Methods. Briefly, in the CUMS model, rats were exposed to two arbitrary mild stressors in each day and the same stressors did not be scheduled in the three consecutive days. Rats in the stress group were randomly exposed to various stressors (e.g. cold, tail pinch and stroboscope) on a daily basis (Fig. 1B), while rats in the control group were handled as usual (daily feeding with adequate food and water). In LH model, rats in the stress group were exposed to an unavoidable, inescapable footshocks for a total of 60 times (intensity 0.85 mA, 15 s average duration, 15 s average interval time, while rats in the control group were placed in the box for the same time without electric shock (Fig. 1C). The learned helpless behaviors of latency to escape and escape failures were then evaluated using an active escape test consisting of 30 trials of escapable footshocks after 5 min of habituation. In CRS model, rats in the stress group were repeatedly placed in a plastic bottle for 6 h (from 09:00 to 15:00) at the same time each day, and both stress and the control groups were deprived of food and water during the restraint stress period (Fig. 1D). In SD model, rats in the stress group were exposed to direct physical contact of Long-Evans (LE) rats for 5 min (Fig. 1E), and then exposed to comprehensive visual, olfactory and auditory exposure of LE rat for 55 min (Fig. 1F), while rats in the control group were placed in empty LE rat home cages for 60 min. The duration of those above four depression models were three weeks. Behavioral tests of the locomotor activity test (LAT), sucrose preference test (SPT), forced swimming test (FST), open field test (OFT), and elevated plus-maze (EPM) have been reported in our previous study [26] and detailed in the Supplementary Methods, and all behavioral tests were conducted from 09:00 to 12:00 P.M. We examined anxiety-like behaviors with the OFT and EPM, and depressive-like behaviors with the SPT and FST.

### Proteomic and metabolomic analysis

The methods of sample preparation for iTRAQ-based proteomics analysis were reported in our previous studies [29]. The right hippocampus of rats was used for proteomics analysis. The left hippocampus of rats was used for metabolomic analysis, and the metabolomic results had been published in our previous studies [26]. For each depression model, two or three protein samples from the stress group or the control group were pooled separately, providing three biological replicates for each group. The pooled samples were digested according to the filter-aided sample preparation (FASP) procedure [30], and labeled using the 8-plex iTRAQ reagent according to the manufacturer’s instructions (Applied Biosystems). Proteins were deemed to be differentially expressed when *p*-value < 0.05 and at least a 1.2-fold change (> 1.20 or < 0.83) relative to the control group. To obtain an overview of the differentially expressed proteins, they were functionally annotated according to biological processes via Kyoto Encyclopedia of Genes and Genomes (KEGG) and Gene Ontology (GO) analysis using DAVID 6.8 (https://david.ncifcrf.gov). KEGG pathway analysis was applied to identify the significantly altered canonical pathways of differentially expressed proteins. In the metabolomic analysis, the details of non-targeted gas chromatography-mass spectrometry (GC-MS) analysis were reported in our previous studies [31]. Metabolic profiling of the processed hippocampus was achieved using an Agilent 7890 A/5975 C GC/MSD System (Agilent Technologies Inc., USA). Metabolites with variable importance in the projection (VIP) values > 1 and FDR < 0.05 were considered significantly different. We used Ingenuity Pathway Analysis (IPA, http://www.ingenuity.com) to summarize the schematic model from the findings of proteomic and metabolic profiling.

### Western blot

Hippocampal tissue was lysed in RIPA buffer with a protease inhibitor cocktail (Roche, Mannheim, Germany). After the KEGG and IPA pathways analysis, we screened key proteins in the pathway related to pathogenesis of depression for further verification. The protein levels of 11 proteins were measured by western blot in hippocampus including protein kinase B (AKT)/ phosphor-AKT, extracellular signal-regulated kinase1/2 (ERK 1/2)/ phosphor-ERK1/2, glutamate receptor 1 (GluA1), mechanistic target of rapamycin (mTOR) and phosphor-mTOR, dual specificity mitogen-activated protein kinase mek-1 (MEK1) and phosphor-MEK1, dual specificity mitogen-activated protein kinase mek-2 (MEK2) and phosphor-MEK2, mitogen-activated protein kinase 13 (P38) and phosphor-P38, ribosomal protein S6 kinase (P70S6K) and phosphor-P70S6K, postsynaptic density protein-95 (PSD95) and phosphor-PSD95, synapsin-1 (Syn1), tropomyosin-related kinase B (TrkB), GAPDH and beta Tubulin.

The proteins were separated on 7.5–10% SDS gels and then transferred to polyvinylidene difluoride membranes (Millipore, Billerica, USA). After blocking in 5% skimmed milk powder for 2 h, incubate the membranes with the primary antibody at 4 °C overnight. After washing with tris buffered saline containing Tween 20 (Beyotime Biotechnology, Shanghai, China), the membranes were incubated with the appropriate concentration of secondary antibody (1:5,000–1:10,000, goat anti-rabbit IgG (H + L)-HPR Conjugate 1706515, Bio-Rad, California, USA) was placed at room temperature for 2 h. The signals were detected with ECL kit (Millipore, Massachusetts, USA), and analyzed with Quantity One software (Bio-Rad, California, USA) [32–35]. The western blotting experiments and catalogue number of the antibody were in the Supplementary Table S1.

### Statistical analyses

The mean ± SEM was used to represent the results of each behavioral test. The results of behavioral tests and western blot protein analysis were compared by SPSS 21.0 (IBM, New York, USA) using independent two-sample Student’s t-tests or non-parametric Mann-Whitney U-tests as appropriate. A *p*-value < 0.05 was considered to be statistically significant.

## Results

### Depressive-like behaviors in the four depression models

Behavioral results showed in Supplementary Fig. S2, and the results had been reported in our previous research [26]. In brief, after screening, four models with 114 rats were included in this study. After stress exposure, the stressed rats were divided into susceptible and resilient groups based on whether their sucrose preference had decreased or not from baseline to endpoint. In the present study, only susceptible rats, designated as the stress group, were used for further analysis, resulting in 68 rats (CUMS/Control =9/8, CRS/Control =8/8, SD/Control=9/8, and LH/Control =10/8). In SPT, the sucrose preference was significantly lower in four stress groups compared to the control groups (Fig. S2E–H). In the FST, immobility times was significantly increased in the four models of depression compared to the control groups (Fig. S2I). In addition, the CUMS model rats showed significantly decreased total distance and rearing frequency in the OFT compared to control group, with no differences in central activity between stress groups and control groups (Fig. S2J–L). In the EPM, CUMS model rats spent less time in the open arms and more time in the closed arms compared to control group (Fig. S2M–P). Taken together, the stress groups of four depression models showed a significant profile of depression-like behaviors, and only CUMS model showed anxiety-like behaviors.

### iTRAQ based proteomic and GC-MS based metabolomic analysis in the hippocampus of four depression models

**Fig. 2:**
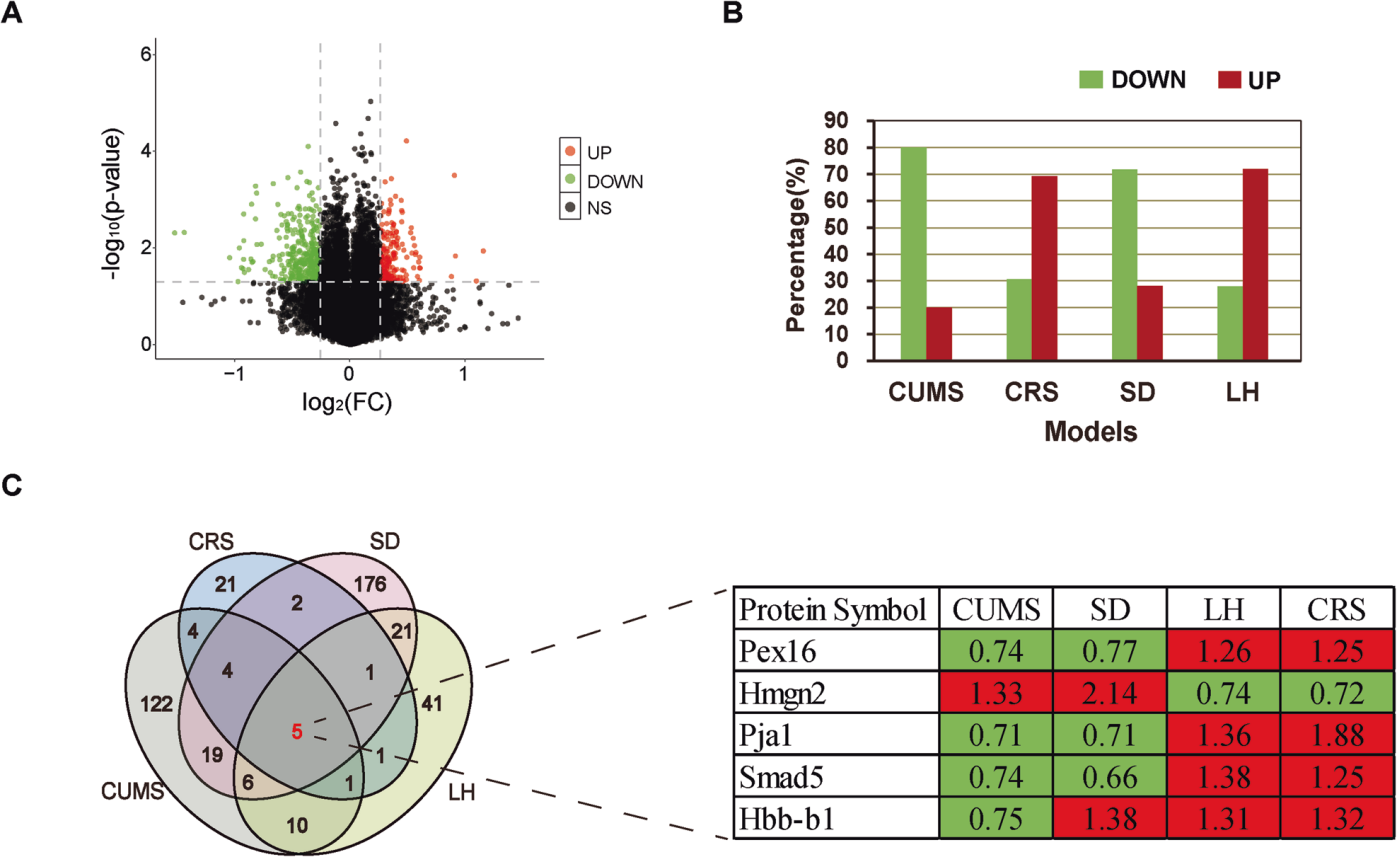
Proteomic analysis results of four depression models. **A** The volcano plot of the iTRAQ analysis. **B** The percentage increase or decrease of differentially expressed proteins for each model. Red, increase; green, decrease. **C** Venn diagram of common and distinct proteins in four depression models, and five commonly expressed proteins and fold change values in four stress-induced models. Red Increase, green, decrease, Pex16 Peroxisomal membrane protein PEX16; Hmgn2, Non-histone chromosomal protein HMG-17, Pjal LOC683077 protein, Smad5 Smad5 protein, Hbb-b1 Hemoglobin subunit beta-1.

A total of 4950 proteins were identified by iTRAQ-based proteomic profiling of hippocampus between stress and control groups in four depression models. The proteomic results of CUMS and SD models were published in our previous studies [29, 36]. Based on the criteria mentioned above, the volcano plot of a total of 529 differentially expressed proteins were identified in four depression models (Fig. 2A), and the percentage increase or decrease of differentially expressed proteins for each model compared to the control groups were shown in Fig. 2B. The details of the differentially expressed proteins in each model were shown in Supplementary Table S2. Among them, the Venn diagram showed 170 differentially expressed proteins in CUMS model, 39 in CRS model; 234 in SD model and 86 in LH model. Interestingly, five commonly expressed proteins (Pex16, Hmgn2, Pja1, Smad5, Hbb-b1) were shared in all of these four stress-induced models (Fig. 2C).

**Fig. 3:**
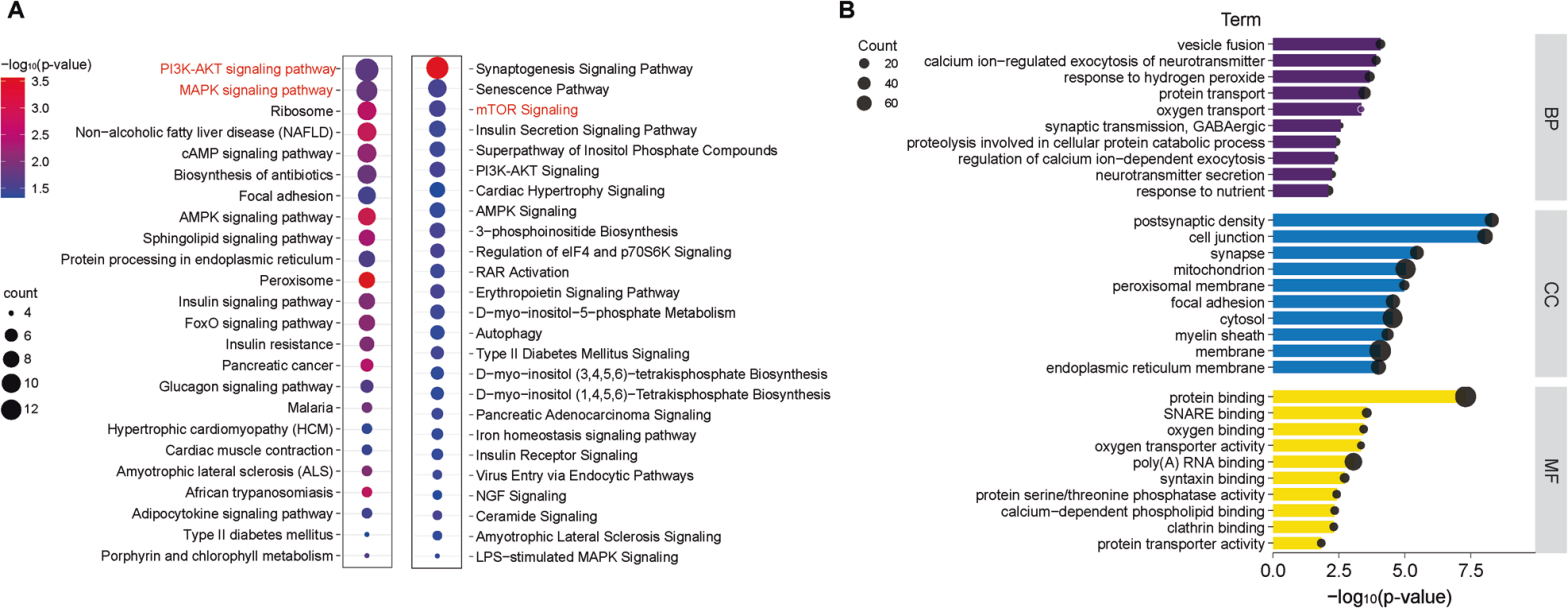
The bioinformatics analysis results of four depression models. **A** The IPA and KEGG pathway analysis of differentially expressed proteins in four depression models. **B** The Gene Ontology (GO) analysis. CUMS Chronic unpredictable mild stress, LH Learned helplessness stress, CRS Chronic restraint stress, SD Social defeat stress, KEGG Kyoto Encyclopedia of Genes and Genomes, IPA Ingenuity Pathway Analysis.

KEGG pathway analysis of 529 differentially expressed proteins in these four depressive-like models identified a total of 24 signaling pathways, of which 16 were significantly different (P < 0.05). At the same time, IPA pathway analysis was performed and a total of 422 pathways were identified, 25 of which were significantly different (FDR P < 0.05; Fig. 3A). Among them, the most prominent and connected pathways were PI3K/AKT, MAPK and mTOR signaling pathways. GO analysis of 529 differentially expressed proteins in four depression models identified into 30 significant GO terms for biological processes (BP), 45 for cellular components (CC), and 18 for molecular function (MF) (Supplementary Table S3), and the top 10 functional analysis results of each category were shown in Fig. 3B. Briefly, in biological processes, the most relevant were vesicle fusion, calcium ion-regulated exocytosis of neurotransmitter, response to hydrogen peroxide, protein transport and oxygen transport; in cellular components, the most relevant were postsynaptic density, cell junction, synapse, mitochondrion and peroxisomal membranes; in molecular function, the most relevant were protein binding, SNARE binding, oxygen binding, oxygen transporter activity and poly RNA binding. The results of hierarchical clustering analysis, and GO analysis of CUMS, CRS, SD and LH models were shown in the Supplementary Fig. S3. The IPA analysis of the differential protein of a single depression model were shown in the Supplementary Fig. S4. Based on the criteria mentioned above, a total of 30, 19, 25 and 24, different metabolites were identified in the CUMS, CRS, SD and LH models. Hippocampus metabolic differences between four rat models of depression were published in our previous research [26].

### The results of western blot of AKT, MAPK and mTOR signaling pathways in four depression models

**Fig. 4:**
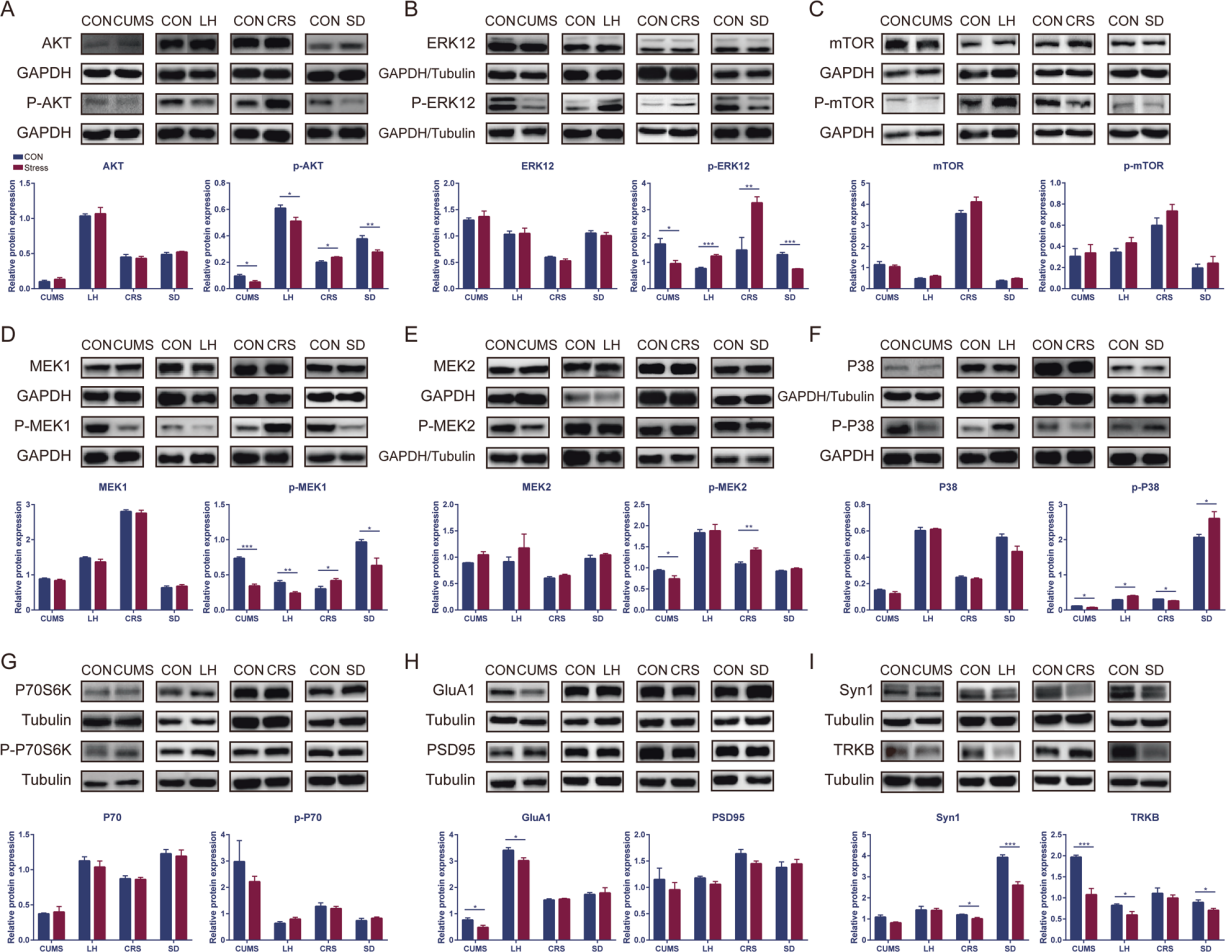
The results of Western blot of hippocampus in four depression models. Representative immunoreactive bands and statistical results showing the protein levels of hippocampus (**A**) AKT and p-AKT, (**B**) ERK12 and p-ERK12, (**C**) mTOR and p-mTOR, (**D**) MEK1 and p-MEK1, (**E**) MEK2 and p-MEK2, (**F**) P38 and p-P38, (**G**) P70S6K and p-P70S6K, (**H**) GluA1 and PSD95, (**I**) Syn1 and TRKB in the stress group compare with control group. All results are represented as means ± SEM; *P < 0.05, **P < 0.01, ***P < 0.001.

Five rats were randomly selected from each the stress and control groups of these four depression models for western blot analysis, and the randomly selected rats were not biased by behavioral differences, as detailed in the Supplementary Fig. S5. Based on AKT, MAPK and mTOR signaling pathways, 11 key proteins (AKT/p-AKT, ERK12/p-ERK12, GluA-1, mTOR/p-mTOR, MEK1/p-MEK1, MEK2/p-MEK2, P38/p-P38, P70S6K/ p-P70S6K, PSD95, Syn1 and TrkB) were chosen to be validated by western blot in four depression models. The results of western blot were shown in the Fig. 4 and detailed in Supplementary Fig. S6. We could find that the AKT and MAPK signaling pathways were significantly altered, while the mTOR signaling pathway was not. Briefly, p-AKT, p-ERK12, p-MEK1 and p-P38 were altered in four depression models (Fig. 4A, B, D, F). Interestingly, p-AKT and p-MEK1 were down-regulated in CUMS, LH and SD models compared to the control groups, while up-regulated in CRS model compared to the control group. p-ERK12 was down-regulated in CUMS and SD models compared to the control groups, while up-regulated in LH and CRS models compared to the control groups. P-P38 was down-regulated in CUMS and CRS models compared to the control groups, while up-regulated in LH and SD models compared to the control groups. TRKB was down-regulated in three depression models (CUMS, LH and SD) compared to the control groups (Fig. 4I). GluA-1 was down-regulated in CUMS and LH models compared to the control groups (Fig. 4H), while Syn1 was down-regulated in CRS and SD models compared to the control groups (Fig. 4I). P-MEK2 was down-regulated in CUMS model compared to the control group, while up-regulated in CRS model compared to the control group (Fig. 4E). There was no difference results in p-mTOR, p-P70S6K and PSD95 in all four depression models between stress groups and control groups (Fig. 4C, G, H).

### Schematic model of AKT and MAPK signaling pathways among four depression models

**Fig. 5:**
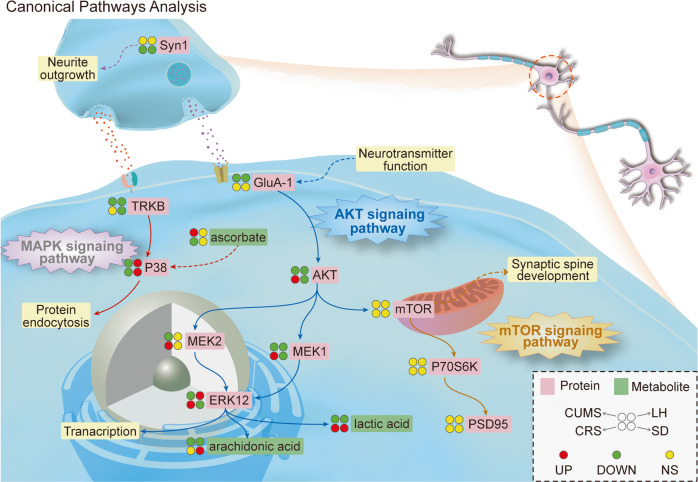
The significantly altered proteins and metabolites in each model were poured into Ingenuity Pathways Analysis (IPA) analysis to find typical pathways and validate key proteins. A schematic model of AKT and MAPK signaling pathways among four depression models in hippocampus. UP Up-regulated, DOWN Down-regulated, NS No significance.

Taken together with the results of the western blot analysis, we found that AKT and MAPK signaling pathways were significantly altered in the hippocampus in all of four stress-induced depression models. We imported the significantly different proteins (Supplementary Table S2) and metabolites [26] from each model, together with Ingenuity Pathways Analysis (IPA) analysis, to find the canonical pathways and validate the key proteins in Fig. 5. The molecular network can be linked by 11 proteins (AKT, ERK12, GluA1, mTOR, MEK1, MEK2, P38, P70S6K, PSD95, SYN1 and TrkB) and 3 metabolites (ascorbate, arachidonic acid and lactic acid), most of which were found to be significantly altered in hippocampus of stress-induced models.

## Discussion

In the present study, proteomic and metabolomic analysis demonstrated that differentially expressed proteins and metabolites in the hippocampus of rats subjected to four stress-induced depression models. According to the pathway analysis of KEGG and IPA, the AKT, MAPK and mTOR signaling pathways were identified as the most prominent pathways. Eight proteins (p-AKT, p-ERK12, GluA1, p-MEK1, p-MEK2, p-P38, Syn1 and TrkB) in the AKT and MAPK signaling pathways were significantly altered in at least one depression model, while three proteins (p-mTOR, p-P70S6K, PSD95) in the mTOR signaling pathway were not altered. Therefore, AKT and MAPK signaling pathways were considered pathways of interest. Previous studies found that CUMS, CRS and LH models of depression reduced the AKT signaling pathway in the hippocampus of mice and that CUMS activated the MAPK signaling pathway [37–40]. Moreover, p-AKT and p-MEK1 were commonly altered in four depression models. Protein phosphorylation plays an important role in signaling processes and regulation of protein function [41] and phosphorylation rapidly changes signaling pathway function and alters the function of proteins associated with the stress-induced depressive disorder [42]. Furthermore, alterations to proteins and functioning via the oxidative phosphorylation pathway within hippocampal synapses had been appreciated in rodent models of stress-induced depression [43]. A schematic model of AKT and MAPK signaling pathway alterations were summarized according to the findings of western blot and our previous metabolomic research in hippocampus of four depression models [26]. To the best of our knowledge, this is the first study to comprehensively identify alterations in hippocampal AKT and MAPK signaling pathways among the four stress-induced depression models.

Although different paradigms had been used to study stress coping, such as genetic, inheritance and environmental, there were commonalities and differences across models [44, 45]. For example, a major similarity between the CSDS and CORT models was that they both exhibited dysregulation of the HPA axis [46, 47] and both showed blunted endocrine response to stress [48, 49]. The difference was that the intrinsic components of susceptibility in CSDS mice were largely dependent on epigenetic factors and early life environment [50, 51], and that the BDNF het-Met variant confers a genetic predisposition to stress-related behaviors in response to applied stressors [52]. Meanwhile, a combined analysis of four genetic mouse models of affective disorders showed that the similarity between these models was highly correlated with regional oxidative metabolism revealed by cytochrome oxidase histochemistry, and different in that the GRi (glucocorticoid receptor) mouse model was characterized by several alterations in oxidative metabolism and altered functional connectivity of the extended amygdala and stress response circuit [53].

Our results revealed differential proteins changes in four depression models. We found inconsistent changes in p-ERK12, GluA1, TrkB, Syn1, and p-P38 proteins among the four depression models. In detail, ERK was coupled to a number of neurotransmitter receptors, including serotonin, adrenergic, dopamine and glutamate receptors, which were highly associated with depression [54]. Chronic stress was associated with decreased protein expression of extracellular signal-regulated kinase 2 (ERK1/2) in the hippocampus. It was known that only phosphorylated proteins exhibit full enzymatic activity, and ERK1/2 phosphorylation was hypothesized to be an intracellular signaling mechanism mediating antidepressant efficacy in patients with depression and in animal models of depression, with supporting evidence coming mainly from studies in rodent or in vitro models [55–57] and postmortem studies of suicidal individuals with depression [58]. GluA1 deficiency exhibits depression-like behavior, and mRNA coding for GluA1 was reduced in rats exposed to chronic stress and also in human hippocampal tissue from depressed patients [59]. In addition, chronic antidepressant treatment in rodents elevated the expression of GluA1 subunit in the hippocampus [60]. The findings that TrkB was important for long-term survival, differentiation, and function of newborn neurons in the adult hippocampus [61], and that neurogenesis played a fundamental role in depression, suggested that discovery of TrkB ligands might open new treatment avenues for this disorder [62]. Synapsin1 (Syn1), as a synapse-associated protein, was expressed in presynaptic membrane and regulates synapse formation [63]. A growing number of studies indicated that alterations of Syn1 were intimately associated with stress-induced depression [64] and that enhancement in Syn1 participates in antidepressant process [64]. Pharmacological blockade of P38 had been suggested to prevent learned helplessness in animal models of depression [65], which was consistent with our results. It had been reported that BDNF has an attenuating effect on the phosphorylation of p38 in primary cell cortex cultures [66]. From the wide spectrum of neuromodulators and cytokines, p38 may be involved in depression-like behavior in sophisticated and interactive ways. The mammalian target of rapamycin (mTOR), as a downstream cascade of BDNF, had been implicated in protein synthesis-dependent synaptic plasticity and can be interrupted in depression [67]. Our results showed the phosphorylation of mTOR or p70S6K in mTOR signaling pathways were not altered in hippocampus of four depression models. The expression of mTOR in depression was controversial and includes increases [68], decreases [37, 69] and no significant changes [70]. While the different experimental conditions may affect the different results. Chandran et al. reported that mTOR signaling pathway alterations only occurred in the amygdala, but not in the hippocampus or frontal cortex in stress-induced depression models [71], which indicated brain region-specific alterations of mTOR. However, these differences between our results may be caused by the different stressors used. The exact nature of this mechanism needs to be further investigated.

On the other hand, we found that the expression of p-MEK1 and p-AKT were consistently expressed in four depression models, down-regulated in the CUMS, LH and SD models and up-regulated in the CRS model. Moreover, the p-MEK2 was down-regulated in CUMS and up-regulated in CRS models. The chronic restraint stress may inhibit the response to a second hit of restraint stress through up-regulated MEK and AKT signaling pathways [72]. However, once these kinases were phosphorylated by upstream MEK1 and MEK2, both ERK1 and ERK2 translocated to the nucleus, where they further phosphorylate target proteins and inhibit or activate transcription of many genes. An earlier study showed that systemic injection of MEK inhibitor resulted in reduced ERK phosphorylation and subsequent depression-like behavior in rats [73]. Preclinical studies had shown that activated AKT promotes resilience to depression-like stress responses [74], whereas high levels of phosphorylated AKT in the hippocampus prolongs contextual and sensitized fear induced by stress [75]. Thus, MEK and AKT cascade signaling may have a critical role in stress-induced depression. We inferred that the potential reason of the opposite expression of MEK and AKT signaling pathways may result from the type of stress when considering the properties of stressors (unpredictable vs. predictable). The stressors in CUMS, SD and LH models were mainly unpredictable, such as unpredictable foot-shock, attack from resident rats and multiple unpredictable in CUMS regime [7, 76, 77]. These findings provided new insights in our understanding of the differential effects of unpredictable and predictable stressors on depressive-like behavior.

In addition, multi-omics analysis also revealed that differentially expressed metabolites (ascorbate, arachidonic acid and lactic acid) commonly found in the hippocampus of four depression models [26], indicating the crosstalk between differentially expressed proteins and metabolites [78]. A recently published meta-analysis revealed that the deficiency of ascorbate (vitamin C) had been linked to depression and cognitive impairment [79]. Moreover, chronic treatment with ascorbate in mice can decrease the hippocampal p38 MAPK phosphorylation, a kinase associated with the release of pro-inflammatory cytokines [80]. Arachidonic acid was the precursor of the Omega-3 fatty acids, such as eicosapentaenoic acid (EPA) and docosahexanoic acid (DHA), which were often associated with antidepressant effects [81, 82]. These effects may be regulated by omega-3 fatty acids through transcriptional regulation by phosphorylation inhibition of ERK pathway [83]. Recent evidence showed that lactate can activate ERK1/2 and AKT pathways [84], and also produced antidepressant effects in animal models by modulating hippocampal neurogenesis [85]. In the present study, we revealed an association between proteins and metabolites in four depression models.

Diverse molecular alterations could converge in similar AKT and MAPK signaling pathways. AKT was a serine/threonine protein kinase that played a central role in the signaling network involving MAPK and mTOR, and which regulates multiple cellular processes including glucose metabolism, apoptosis, cell proliferation, transcription and cell migration [86]. Depression was associated with cellular impairments in neuronal function, which may consequently manifest as abnormalities in neuroplasticity [87]. AKT had received extensive consideration in recent years for its possible involvement in psychiatric conditions, and AKT deletion evoked a change in behavior reflecting depression [87, 88]. Furthermore, the subcellular integration of the dopamine and serotonin neurotransmission was regulated by AKT, which may contribute to the development of several psychiatric conditions such as MDD [89]. The mitogen-activated protein kinase (MAPK) pathway played an important role in signal transduction by converting extracellular stimuli into a wide range of cellular responses including stress response, inflammatory response, differentiation, and survival [90]. The MAPK pathway responded to excitatory glutamatergic signaling controlling synaptic plasticity and higher brain processes such as learning and memory. Importantly, this pathway was related to neuropathological processes including depression [91]. Increasing evidence supported a pivotal role of the mitogen-activated protein kinase (MAPK) in the pathogenesis, symptomatology, and treatment of depression, in particular the extracellular signal-regulated kinase (ERK) subclass of MAPKs [54]. Furthermore, chronic administration of lithium or valproate, mood stabilizers used in the treatment of manic depression, stimulated the MAPK pathway in the rat hippocampus [92]. Overall, our results supported the potential involvement of proteins and metabolites in the altered signaling pathways in hippocampus of four depression models.

There were several limitations in this study. First, only a number of classic molecular of depression-related proteins involve in AKT, MAPK and mTOR signaling pathways were detected by western blot, with no evidence of functional effects of cell transduction. Future research should incorporate a broader analysis including the other altered proteins and pathways. Second, although most depression model studies have been conducted with male rats, this would cause a gender bias [93]. Further investigation should be done in both male and female rats. Third, we did not explore the synaptic neuroplasticity, cell proliferation, cell migration, and apoptosis, which were associated with AKT or MAPK signaling pathways. Further studies should be performed to concentrate on these targets. Fourth, this study was performed in the whole hippocampus and not the dorsal or ventral hippocampus, which is an important distinction since the different hippocampal subfields have different neural projections and functions [94]. Fifth, the SPT to assess depression-like behaviors were based on novelty, therefore, consecutive tests may affect the observed results. Sixth, sucrose was given to the rats prior to multi-omics tests, which may cause alterations in metabolomics and proteomics [95–97]. Finally, the complex features of depression cannot be totally captured by animal models. Thus, it is crucial to translate our present findings from animals to humans in future studies.

In summary, we integrated proteomic and metabolomic analysis of multiple stress-induced depression models in the hippocampus of rats, which were mainly involved in AKT and MAPK signaling pathways. Then, nine proteins in AKT and MAPK pathways were identified to be altered by western blot. Finally, AKT and MAPK pathways molecular network were demonstrated when combining with altered metabolites in hippocampus. These findings advance our understanding of the potential pathophysiology and heterogeneity of depression as manifested in the hippocampus, and it could facilitate the development of personalized medicine based on these novel therapeutic targets for depression.

## Supplementary information

Supplementary Materials

## Data Availability

The Proteomic data were deposited in the Integrated Proteome Resources (iProX) (https://www.iprox.cn/page/home.html; project ID: IPX0006454000).
