# Supplementary material for: AKT and MAPK signaling pathways in hippocampus reveals the pathogenesis of depression in four stress-induced models
Source: Transl Psychiatry. 2023 Jun 12;13:200. doi: 10.1038/s41398-023-02486-3 (PMC10261007; doi:10.1038/s41398-023-02486-3)
Supplement: Supplementary file 1 — Supplementary Materials [file 41398_2023_2486_MOESM1_ESM.docx]

**AKT and MAPK signaling pathways in hippocampus reveals the pathogenesis of depression in four stress-induced models**

Xuemei Li, Ph.D.^1,2,†^, Teng Teng, Ph.D.^1,2,^^†^, Wei Yan, Ph.D.^3^, Li Fan, Ph.D.^2,4^, Xueer Liu, M.D.^2,4^, Gerard Clarke, Ph.D.^5,6^, Dan Zhu, Ph.D.^4^, Yuanliang Jiang, M.D.^1,2^, Yajie Xiang, Ph.D.^2,4^, Ying Yu, Ph.D.^2,4^, Yuqing Zhang, Ph.D.^2,7^, Bangmin Yin, Ph.D.^1,2^, Lin Lu, Ph.D.^3,*^, Xinyu Zhou, Ph.D.^1,2,*^, Peng Xie, M.D.^2,4,*^

1 Department of Psychiatry, The First Affiliated Hospital of Chongqing Medical University, Chongqing, China;

2 NHC Key Laboratory of Diagnosis and Treatment on Brain Functional Diseases, The First Affiliated Hospital of Chongqing Medical University, Chongqing, China;

3 Peking University Sixth Hospital, Peking University Institute of Mental Health, NHC Key Laboratory of Mental Health (Peking University), National Clinical Research Center for Mental Disorders (Peking University Sixth Hospital), Beijing, China;

4 Department of Neurology, The First Affiliated Hospital of Chongqing Medical University, Chongqing, China;

5 Department of Psychiatry and Neurobehavioural Science, University College Cork, Cork, Ireland;

6 APC Microbiome Ireland, University College Cork, Cork, Ireland;

7 Department of Neurology, The Second Affiliated Hospital of Chongqing Medical University, Chongqing, China;

†These authors contributed equally to the manuscript.

* Corresponding authors:

Peng Xie,

Department of Neurology, The First Affiliated Hospital of Chongqing Medical University,

1 Youyi Road, Yuzhong District, Chongqing, China

E-mail: xiepeng@cqmu.edu.cn

Xinyu Zhou,

Department of Psychiatry, The First Affiliated Hospital of Chongqing Medical University 1 Youyi Road, Yuzhong District,

Chongqing, China

E-mail: zhouxinyu@cqmu.edu.cn

Lin Lu,

Peking University Sixth Hospital/Peking University Institute of Mental Health, 51 Huayuanbei Road, Haidian District,

Beijing, China.

E-mail: linlu@bjmu.edu.cn

| Methods | The detail of chronic unpredictable mild stress (CUMS), learned helplessness (LH), chronic restraint stress (CRS), and social defeat (SD) paradigm and behavior tests. | Page 4 |
| --- | --- | --- |
| Figure S1 | A flowchart of the molecular profiling methods. | Page 7 |
| Figure S2 | The results of the behavioral tests in four depression models. | Page 8 |
| Figure S3 | The results of hierarchical clustering analysis and GO analysis of CUMS, CRS, SD and LH models. | Page 9 |
| Figure S4 | The IPA analysis of the differential protein of four stress-induced depression models. | Page 10 |
| Figure S5 | Behavioral results of randomly selected protein validation rats. | Page 11 |
| Figure S6 | The results of western blot of 11 key proteins. | Page 12 |
| Table S1 | The brand and catalogue number of the antibody used in Western blot. | Page 13 |
| Table S2 | The details of the differentially expressed proteins of iTRAQ-based proteomic in four stress-induced depression models. | Page 14 |
| Table S3 | The detailed information of GO analysis of 529 differentially expressed proteins in the four stress-induced depression models. | Page 49 |

**Supplementary Materials**

**Supplementary Methods**

**The details of Chronic Unpredictable Mild Stress (CUMS), Learned Helplessness (LH), Chronic Restraint Stress (CRS), and Social Defeat (SD) paradigm and behavior tests have been reported in our previous research (Liu L et al., 2018).**

**CUMS model**

The CUMS model simulated a typical depression manifestation in a rat through a series of chronic, mild, unpredictable stress stimuli. Rats in the stress group were randomized to different stimuli every day for 3 weeks, while rats in the control group received only regular handling. In the CUMS model, rats were exposed to two arbitrary mild stressors in each day and the same stressors did not be scheduled in the three consecutive days. The pressure source in the CUMS protocol was based on previously published protocols (Liu L et al., 2018), including 4 hours of restraint, 24 hours of deprivation of water/food, 1 hour of cold stimulation at 4 °C, 1 minute of tail-tailing, swimming in water at 18 °C for 5 minutes, turn on the lights for 12 hours, turn off the lights for 3 hours, the stroboscope was irradiated for 12 hours, reverse the light/dark cycle for 24 hours, the cages were tilted for 24 hours, the cages were crowded for 24 hours, and the bedding was wet for 24 hours. The procedure for the CUMS model was shown in Figure 1B.

**LH model**

In the LH model, the experimental rats were placed on one side of a shuttle box (Shandong Academy of Medical Sciences, China) and exposed to an inevitable footshocks for a total of 60 times (intensity 0.85 mA, 15 s average duration, 15 s average interval time). Rats in the control group were placed in the box for the same time, but there was no electric shock. The learned helpless behaviors of latency to escape and escape failures were then evaluated using an active escape test consisting of 30 trials of escapable footshocks (0.8 mA intensity, 10 s maximum duration, 30 s average inter-trial interval) after 5 min of habituation. The LH program has been reported elsewhere (Zhou X et al., 2017).The procedure lasted for 3 weeks, as shown in Figure 1C.

**CRS model**

The CRS model was used to simulate a subtype of depression caused by restraint stress for 3 weeks, which was an important risk factor for the etiology of depression. Rats in the experimental group were repeatedly placed in a modified plastic bottle (550 ml volume water bottle, Nongfu Spring Company Limited, Hang Zhou, China) for 6 hours (from 09:00 to 15:00) at the same time each day. Both the stress group and the control group were deprived of food and water during the restraint stress period. Detailed operational specifications (Liu L et al., 2016) were reported in our previous study and procedure was shown in Figure 1D.

**SD model**

The SD model was modified by the occupant-invader model (Koolhaas JM et al., 2013) to simulate depression in rats due to stress. The social defeat model consists of two consecutive periods (60 minutes total) in the home cage of LE rats. During period I (5 min), the rats were subjected to physical confrontation. In the period I, when the interaction becomes too strong or once the SD rat surrenders or appears supine within 5 seconds, the SD rat was placed in a small wire mesh protective cage (10 cm × 10 cm × 15 cm). In period II, the remaining 55 minutes were spent at LE's home. Therefore, the use of wire mesh cages was allowed for comprehensive visual, olfactory and auditory exposure of LE rat without direct physical contact. Rats in the control group were placed in empty LE rat home cages for 60 minutes. The SD model has been reported elsewhere (Liu YY et al., 2017) and procedure lasted for 3 weeks, as shown in Figure 1E and Figure 1F.

**Behavioral tests**

The locomotor activity test (LAT), sucrose preference test (SPT), forced swimming test (FST), open field test (OFT), and elevated plus -maze (EPM) test were conducted as previously described (Liu et al., 2018). Briefly, LAT was used to identify the level of free activity in rats prior to experimentation. The athletic activity index was the distance (in centimeters) that was traveled in the open field device over the five-minute test period. The SPT was administered once a week. Sucrose preference was calculated as sucrose intake/total liquid intake (water + sucrose). The SPT test lasted for one hour and was used to assess the anhedonia. The body weight of rats was measured weekly at the end of the SPT test. The total immobility time in the five-minute FST test was recorded and used to assess depression-like behavior. In the five-minute OFT test, locomotor activity, central activity, and rearing frequency were measured to assess spatial exploration behavior. The number of times rats entered the open and closed arms and the time spent in each arm were recorded over the five-minute EPM test to assess anxiety-like behavior. Movements in the LAT, FST, OFT, and EPM were recorded and analyzed using a video surveillance system (SMART, Panlab SL, Barcelona, Spain).

**Reference**

Koolhaas JM, Coppens CM, de Boer SF, Buwalda B, Meerlo P, Timmermans PJ (2013), The resident-intruder paradigm: a standardized test for aggression, violence and social stress. J Vis Exp:e4367.

Liu L, Zhou X, Zhang Y, Liu Y, Yang L, Pu J, Zhu D, Zhou C, et al. (2016), The identification of metabolic disturbances in the prefrontal cortex of the chronic restraint stress rat model of depression. Behav Brain Res 305:148-156.

Liu L, Zhou X, Zhang Y, Pu J, Yang L, Yuan S, Zhao L, Zhou C, et al. (2018), Hippocampal metabolic differences implicate distinctions between physical and psychological stress in four rat models of depression. Transl Psychiatry 8:4.

Liu YY, Zhou XY, Yang LN, Wang HY, Zhang YQ, Pu JC, Liu LX, Gui SW, et al. (2017), Social defeat stress causes depression-like behavior with metabolite changes in the prefrontal cortex of rats. PLoS One 12:e0176725.

Zhou X, Liu L, Zhang Y, Pu J, Yang L, Zhou C, Yuan S, Zhang H, et al. (2017), Metabolomics identifies perturbations in amino acid metabolism in the prefrontal cortex of the learned helplessness rat model of depression. Neuroscience 343:1-9.

**Supplementary Figure S1:** A flowchart of the molecular profiling methods.
**Supplementary Figure S2:** The results of the behavioral tests in four depression models.

Behavioral results have been reported in our previous research (Liu L et al., 2018). There were no significant differences in weight or sucrose preference test (SPT) results between the Stress and Control groups at baseline (Figure S2A-H). Body weight gain in the CUMS, CRS and SD groups was significantly lower than in the control group (Figure S2A,C,D). Similarly, sucrose preference was lower in the stress-induced group than in the control group (Figure S2E–H). In the forced swimming test (FST), the immobility time in the stress-induced group was longer than that in the control group (Figure S2I), which indicates that depression-like behavior was aggravated in the CUMS, LH, CRS and SD groups. Moreover, the CUMS model rats showed a significant decrease in total distance and rearing frequency in the open field test (OFT) (Figure S2J,L), with no significant effect in central activity. In the elevated plus -maze (EPM), CUMS model rats spent less time in the open arms and more time in the closed arms (Figure S2N,P). However, the LH, CRS and SD groups were no significant differences in the total distance, central activity, or rearing frequency in the OFT (Figure S2J–L). Similarly, there were no differences in the total number of entries or the time spent in the open and closed arms between the two groups in the EPM (Figure S2M–P). All results are represented as means ± SEM; *P< 0.05, **P< 0.01, ***P< 0.001.

**Supplementary Figure S3：**The results of hierarchical clustering analysis and GO analysis of CUMS, CRS, SD and LH models.

The bioinformatics analysis of differential proteins in each depression model. There were 170 differentially expressed proteins in CUMS model, 39 in CRS model; 234 in SD model and 86 in LH model. (A) The hierarchical clustering analysis, (B) The GO analysis of each model, and in the CUMS and SD models top ten functional analysis results of each category (Biological Process, Cellular Component, and Molecular Function) were listed, and all the significant GO terms were listed of CRS and LH models.

**Supplementary Figure S4:** The IPA analysis of the differential protein of four stress-induced depression models.

The Ingenuity Pathways Analysis (IPA) identified differentially regulated canonical pathways in CUMS model (A), LH model (B), CRS model (C) and SD model (D). The top ten significant pathway of CUMS, SD, LH model were listed, and all the six significant pathway of CRS model were listed.

**Supplementary Figure S5:** Behavioral results of randomly selected protein validation rats.

We compared differences in depression-like behavior between groups using t-test or Mann-Whitney U-test. CUMS-WB, CUMS rats for protein validation; SD-WB, SD rats for protein validation; LH-WB, LH rats for protein validation; CRS-WB, CRS rats for protein validation; *P< 0.05, **P< 0.01.

**Supplementary Figure S6:** The results of western blot of 11 key proteins.

The bands of western blot of 11 key proteins (AKT/p-AKT, ERK12/p-ERK12, GluA-1, mTOR/p-mTOR, MEK1/p-MEK1, MEK2/p-MEK2, P38/p-P38, P70S6K/ p-P70S6K, PSD95, Syn1 and TrkB) in CUMS model, LH model, CRS model and SD model.

**Supplementary Table S1:** The brand and catalogue number of the antibody used in Western blot.

| Antibodies | Source | Identifier | Dilution |
| --- | --- | --- | --- |
| Anti-Akt (pan) | Cell Signaling Technology | 4685 | 1:1000 |
| Anti-Phospho-Akt (Ser473) | Cell Signaling Technology | 4060 | 1:1000 |
| Anti-ERK1 + ERK2 | Abcam | ab17942 | 1:1000 |
| Anti-ERK1 + ERK2 (phospho T202 + Y204) | Abcam | ab214362 | 1:1000 |
| Anti-Glutamate Receptor 1 | Abcam | ab183797 | 1:1000 |
| Anti-mTOR | Cell Signaling Technology | 2983 | 1:1000 |
| Anti-mTOR (phospho S2448) | Abcam | ab109268 | 1:1000 |
| Anti-MEK1 | Abcam | ab32091 | 1:1000 |
| Anti-MEK1 (phospho S298) | Abcam | ab96379 | 1:1000 |
| Anti-MEK2 | Abcam | ab32517 | 1:2500 |
| Anti-MEK2 (phospho T394) | Abcam | ab30622 | 1:1000 |
| Anti-p38 | Abcam | ab31828 | 1:1000 |
| Anti-p38 (phospho T180 + Y182) | Abcam | ab4822 | 1:1000 |
| Anti-p70 S6 Kinase Antibody | Cell Signaling Technology | 9202 | 1:1000 |
| Anti-Phospho-p70 S6 Kinase (Thr389) | Cell Signaling Technology | 9205 | 1:1000 |
| Anti-PSD95 | Abcam | ab76115 | 1:1000 |
| Anti-Synapsin-1 | Cell Signaling Technology | 5297 | 1:1000 |
| Anti-TrkB | Abcam | ab18987 | 1:1000 |
| Anti-GAPDH | Abcam | ab8245 | 1:10000 |
| Anti-beta Tubulin | Abcam | ab6046 | 1:10000 |

**Supplementary Table S2:** The details of the differentially expressed proteins of iTRAQ-based proteomic in four stress-induced depression models.

| Accession | Description | Symbol | CUMS | | LH | | CRS | | SD | |
| --- | --- | --- | --- | --- | --- | --- | --- | --- | --- | --- |
|  |  |  | Fold change | P value | Fold change | P value | Fold change | P value | Fold change | P value |
| Overlap across all models | | | | | | | | | | |
| A0A0G2JTW9 | Hemoglobin subunit beta-1 | Hbb-b1 | 0.747669526 | 0.00524067 | 1.308887402 | 0.0286967 | 1.32429167 | 0.00834464 | 1.381073987 | 0.0110491 |
| A0A0G2JWT6 | Peroxisomal membrane protein PEX16 | Pex16 | 0.737947613 | 0.0393302 | 1.257457997 | 0.0425189 | 1.254071972 | 0.0165772 | 0.766299076 | 0.0132572 |
| P18437 | Non-histone chromosomal protein HMG-17 | Hmgn2 | 1.331791007 | 0.00617552 | 0.739092691 | 0.00207844 | 0.723962653 | 0.0181932 | 2.137817989 | 0.048046 |
| Q66HF7 | LOC683077 protein | Pja1 | 0.707777949 | 0.0363042 | 1.364017277 | 0.00166749 | 1.884876984 | 0.0145989 | 0.712234444 | 0.0103817 |
| Q6IRI8 | Smad5 protein | Smad5 | 0.743724567 | 0.0124032 | 1.378626967 | 0.047958 | 1.249314865 | 0.0229765 | 0.656051694 | 0.0436269 |
| Overlap across three models | | | | | | | | | | |
| A0A0G2JU85 | Dendrin | Ddn | 0.552422341 | 0.0012322 | … | … | 1.264128228 | 0.0206289 | 0.718125733 | 0.00415821 |
| A0A0H2UI11 | 60S ribosomal protein L39 | Rpl39 | 0.347684584 | 0.00484244 | … | … | 1.873575271 | 0.000312801 | 0.742255771 | 0.0439421 |
| P51869 | Cytochrome P450 4F4 | Cyp4f4 | 0.702907008 | 0.00194495 | … | … | 1.482340262 | 0.013615 | 0.557077942 | 0.0190227 |
| Q8R553 | Calsyntenin-3 | Clstn3 | 0.526740297 | 0.0170996 | … | … | 1.28752532 | 0.0384444 | 0.525018055 | 0.0268667 |
| Q5XIB1 | Protein TSSC4 | Tssc4 | 0.791795961 | 0.00779007 | 1.215401336 | 0.0187221 | 2.229282788 | 0.0114746 | … | … |
| D3ZZN4 | 60S ribosomal protein L35a | NA | 0.801653553 | 0.0121195 | 0.830828246 | 0.019079 | … | … | 1.465930758 | 0.0256353 |
| D4A709 | Tubulin, gamma complex-associated protein 6 | Tubgcp6 | 0.554027465 | 0.0196866 | 1.320438 | 0.0486944 | … | … | 0.651899054 | 0.0144958 |
| O08589 | Phospholemman | Fxyd1 | 0.686395386 | 0.00445243 | 1.23951969 | 0.0332012 | … | … | 0.808749762 | 0.02993 |
| Q63327 | Myelin-associated oligodendrocyte basic protein | Mobp | 0.832747138 | 0.0136881 | 0.773646942 | 0.00287149 | … | … | 1.445508895 | 0.00379598 |
| Q642B5 | Armadillo repeat containing, X-linked 2 | Armcx2 | 0.749338121 | 0.00810088 | 1.292720725 | 0.00750895 | … | … | 0.758882057 | 0.00423495 |
| Q7TP07 | Da1-12 | NA | 0.737358112 | 0.0200812 | 1.233826284 | 0.022916 | … | … | 0.784916741 | 0.032204 |
| A1A5P9 | Melanoma-associated antigen E1 | Magee1 | … | … | 1.48644985 | 0.0123331 | 1.840672504 | 0.0387792 | 0.52710035 | 0.00703248 |
| Overlap across two models | | | | | | | | | | |
| C6JUM5 | Protocadherin gamma C5 | Pcdhgc5 | 0.742406295 | 0.00026766 | … | … | 1.20290348 | 0.0362555 | … | … |
| D4ADG2 | Iron-sulfur cluster assembly factor IBA57 | Iba57 | 0.706742015 | 0.0163449 | … | … | 0.712667387 | 0.0156269 | … | … |
| Q6MG82 | Proline-rich transmembrane protein 1 | Prrt1 | 0.729049464 | 0.0325279 | … | … | 0.736175839 | 0.0335747 | … | … |
| P11517 | Hemoglobin subunit beta-2 | N/A | 0.627386632 | 0.0380749 | … | … | 1.363702473 | 0.00285527 | … | … |
| A0A0G2K0W0 | Geranylgeranyl pyrophosphate synthase | Ggps1 | 0.763594435 | 0.0203905 | … | … | … | … | 0.821527039 | 0.0154031 |
| A0A0G2K913 | 5'-AMP-activated protein kinase subunit gamma-1 | Prkag1 | 0.831481121 | 0.00353789 | … | … | … | … | 0.818013277 | 0.0161275 |
| B2GV65 | HIG1 domain family, member 2A | Higd2a | 1.281038322 | 0.0082481 | … | … | … | … | 1.225597725 | 0.00176513 |
| D3ZIP8 | Uncharacterized protein | N/A | 0.748125482 | 0.00562095 | … | … | … | … | 1.200359064 | 0.00441516 |
| D3ZKR8 | Protein kish | Tmem167a | 1.269456039 | 0.025103 | … | … | … | … | 0.8269152 | 0.0233721 |
| D3ZW27 | Mitogen-activated protein kinase kinase kinase 5 | Map3k5 | 0.812031422 | 0.0346599 | … | … | … | … | 0.741255632 | 0.0472866 |
| F1LPQ4 | Phospholipase | Pld2 | 0.746862256 | 0.0311884 | … | … | … | … | 0.672750835 | 0.002148 |
| F1M6E5 | NAC alpha domain-containing | Nacad | 0.71619159 | 0.0288345 | … | … | … | … | 0.78974696 | 0.015655 |
| F1MAL5 | Insulin receptor substrate 2 | Irs2 | 0.831412386 | 0.0015949 | … | … | … | … | 0.720964093 | 0.0468568 |
| G3V6R0 | Amino acid transporter | Slc1a2 | 0.671369512 | 0.0468449 | … | … | … | … | 0.484152463 | 0.0158302 |
| O88778 | Protein bassoon | Bsn | 0.550398774 | 0.0168507 | … | … | … | … | 0.726369648 | 0.000563535 |
| P01355 | Cholecystokinin | Cck | 0.832833474 | 0.028923 | … | … | … | … | 0.787667458 | 0.00526986 |
| P36877 | Serine/threonine-protein phosphatase 2A 55 kDa regulatory subunit B beta isoform | Ppp2r2b | 0.726364178 | 0.0243265 | … | … | … | … | 0.717733008 | 0.0392674 |
| Q5FVL9 | Fam103a1 protein | Fam103a1 | 1.216367336 | 0.0258283 | … | … | … | … | 1.247437043 | 0.0273786 |
| Q63186 | Translation initiation factor eIF-2B subunit delta | Eif2b4 | 0.809918844 | 0.0218523 | … | … | … | … | 0.660604013 | 0.0485969 |
| Q641Z8 | Peflin | Pef1 | 0.801292891 | 0.010279 | … | … | … | … | 0.829501106 | 0.0434649 |
| Q7TQN4 | Nuclear factor kappaB subunit p65 | Rela | 0.69452007 | 0.0223868 | … | … | … | … | 0.709652144 | 0.00507259 |
| Q920J3 | Coronin-6 | Coro6 | 0.784044227 | 0.0175585 | … | … | … | … | 0.661835759 | 0.0225201 |
| Q9ERM7 | Secretory carrier-associated membrane protein | N/A | 0.670884426 | 0.0451925 | … | … | … | … | 0.519897521 | 0.0254498 |
| A0A0G2JSW3 | Globin a4 | Hbb | 0.685387094 | 0.00341491 | 1.312521679 | 0.00562877 | … | … | … | … |
| A0A0G2JTX2 | PRA1 family protein | Praf2 | 0.56946202 | 0.000726417 | 1.264716541 | 0.0195337 | … | … | … | … |
| A0A0G2JUZ5 | Glycine cleavage system P protein | Gldc | 0.801308899 | 0.00430653 | 1.281608891 | 0.000365279 | … | … | … | … |
| A0A0G2K2Y3 | Aminopeptidase | Erap1 | 0.671594739 | 0.0292827 | 1.212568283 | 0.021347 | … | … | … | … |
| A0A0G2K583 | S-methyl-5'-thioadenosine phosphorylase | Mtap | 0.687493542 | 0.000347963 | 1.291721889 | 0.00482529 | … | … | … | … |
| B5DFI3 | AP complex subunit sigma | Ap1s1 | 0.811859721 | 0.0400919 | 0.808137639 | 0.0486799 | … | … | … | … |
| D4ACB6 | Pleckstrin and Sec7 domain containing 2 (Predicted) | Psd2 | 0.750797286 | 0.00588656 | 1.27382779 | 0.00101007 | … | … | … | … |
| Q4KLI0 | SWI/SNF-related matrix-associated actin-dependent regulator of chromatin subfamily B member 1 | Smarcb1 | 0.782629291 | 0.000822649 | 1.233474315 | 0.0052357 | … | … | … | … |
| Q5XI90 | Dynein light chain Tctex-type 3 | Dynlt3 | 0.715046213 | 0.00924047 | 1.247201202 | 0.00564313 | … | … | … | … |
| Q9JJP0 | Sodium-dependent phosphate transporter 1 | Slc20a1 | 0.738572344 | 0.00157031 | 0.782192966 | 0.00153932 | … | … | … | … |
| F1LQ27 | Family with sequence similarity 98, member C | Fam98c | … | … | … | … | 1.20685812 | 0.0153212 | 0.809621815 | 0.0406256 |
| Q91XQ2 | Laforin | Epm2a | … | … | … | … | 1.232822506 | 0.0470761 | 0.761723511 | 0.0128353 |
| Q568Y7 | Noelin-2 | Olfm2 | … | … | 1.365525877 | 0.0499065 | 1.304517569 | 0.0299419 | … | … |
| Q80XF7 | Gap junction gamma-2 protein | Gjc2 | … | … | 1.402986228 | 0.0449585 | … | … | 0.708902407 | 0.0396967 |
| A0A096MKD9 | Tripartite motif-containing protein 46 | Trim46 | … | … | 1.227517219 | 0.0485631 | … | … | 0.742158605 | 0.0173249 |
| A0A0G2K5E8 | Collagen, type I, alpha 2 | NEWGENE_621351 | … | … | 0.720559198 | 0.0220659 | … | … | 1.47262989 | 0.00887455 |
| A0A0G2K654 | Histone cluster 1 H1 family member c | Hist1h1c | … | … | 0.791157296 | 0.0202707 | … | … | 1.510741165 | 0.0193975 |
| B1WBZ7 | GPN-loop GTPase | Gpn1 | … | … | 1.293849659 | 0.049789 | … | … | 0.756248532 | 0.007139 |
| B2GV96 | Coiled-coil domain-containing 115 | Ccdc115 | … | … | 1.321748307 | 0.00468631 | … | … | 0.615009976 | 0.0191148 |
| D3ZHP7 | Serine/threonine-protein kinase ULK3 | Ulk3 | … | … | 1.243467014 | 0.0168685 | … | … | 0.826478565 | 0.0126076 |
| D4A3L3 | EGF-like-domain, multiple 5 (Predicted) | Megf9 | … | … | 0.808316737 | 0.010495 | … | … | 1.246298634 | 0.0242332 |
| D4A4P8 | Protein tweety homolog | Ttyh2 | … | … | 1.221932705 | 0.0109069 | … | … | 0.696769531 | 0.0465535 |
| D4ADA1 | Fibrinogen C domain containing 1 (Predicted) | Fibcd1 | … | … | 1.312724304 | 0.0128274 | … | … | 0.687022737 | 0.0131295 |
| F1LQI2 | Roundabout homolog 1 | Robo1 | … | … | 1.252415184 | 0.0153068 | … | … | 0.643377333 | 0.0043157 |
| F1LR84 | Neuronal pentraxin-2 | Nptx2 | … | … | 1.234355418 | 0.0427368 | … | … | 0.698222158 | 0.0132393 |
| F1LSB5 | Uncharacterized protein | N/A | … | … | 1.215715755 | 0.00450059 | … | … | 0.824224363 | 0.0169915 |
| G3V8P3 | Cadherin EGF LAG seven-pass G-type receptor 2 | Celsr2 | … | … | 1.314017195 | 0.0159721 | … | … | 0.82134019 | 0.0136877 |
| M0R7B4 | Histone cluster 1 H1 family member d | Hist1h1d | … | … | 0.746142318 | 0.0184832 | … | … | 1.293612628 | 0.00379499 |
| O55145 | Fractalkine | Cx3cl1 | … | … | 1.246549697 | 0.0102934 | … | … | 0.738033309 | 0.042082 |
| P0C644 | Inositol hexakisphosphate and diphosphoinositol-pentakisphosphate kinase 1 | Ppip5k1 | … | … | 1.3028149 | 0.0423291 | … | … | 0.542610669 | 0.027805 |
| P41350 | Caveolin-1 | Cav1 | … | … | 0.798872782 | 0.00624139 | … | … | 1.295357585 | 0.0488901 |
| Q498C8 | Protein RER1 | Rer1 | … | … | 1.382690758 | 0.029447 | … | … | 0.719244175 | 0.0234364 |
| Q5U201 | Protein AMN1 homolog | Amn1 | … | … | 1.226716589 | 0.00627231 | … | … | 0.629057065 | 0.0147326 |
| Q9JIR4 | Regulating synaptic membrane exocytosis protein 1 | Rims1 | … | … | 1.276197014 | 0.0326777 | … | … | 0.805864007 | 0.0408553 |
| Model special | | | | | | | | | | |
| A0A096MJN8 | CKLF-like MARVEL transmembrane domain-containing 5 | Cmtm5 | 0.720142181 | 0.0326178 | … | … | … | … | … | … |
| A0A096XKT9 | NADH-ubiquinone oxidoreductase chain 5 | ND5 | 0.831618802 | 0.00958115 | … | … | … | … | … | … |
| A0A0A0MY08 | Junctophilin | Jph4 | 0.802068882 | 0.0348977 | … | … | … | … | … | … |
| A0A0G2JV26 | Protein phosphatase 4, regulatory subunit 4 | Ppp4r4 | 0.804647674 | 0.0206294 | … | … | … | … | … | … |
| A0A0G2JXH4 | Unconventional myosin-IXa | Myo9a | 0.247714386 | 0.0151053 | … | … | … | … | … | … |
| A0A0G2K0B6 | Clathrin coat assembly protein AP180 | Snap91 | 1.206568774 | 0.0299405 | … | … | … | … | … | … |
| A0A0G2K0P2 | Mothers against decapentaplegic homolog | Smad2 | 0.652909644 | 0.00616434 | … | … | … | … | … | … |
| A0A0G2K0T6 | Gamma-synuclein | Sncg | 0.804231043 | 0.0294378 | … | … | … | … | … | … |
| A0A0G2K1W1 | RAB11 family-interacting protein 5 | Rab11fip5 | 0.565694936 | 0.00248751 | … | … | … | … | … | … |
| A0A0G2K6M8 | DEP domain-containing 5 | Depdc5 | 1.293929034 | 0.0434064 | … | … | … | … | … | … |
| A0A0G2K9N3 | DEAD (Asp-Glu-Ala-Asp) box polypeptide 3 | Ddx3 | 0.81708238 | 0.0106836 | … | … | … | … | … | … |
| A0A0H2UH93 | MAM domain-containing glycosylphosphatidylinositol anchor protein 2 | Mdga2 | 1.333052214 | 0.0463229 | … | … | … | … | … | … |
| A0A0H2UHD5 | Leucine-rich repeat transmembrane protein FLRT3 | Flrt3 | 1.253200908 | 0.011684 | … | … | … | … | … | … |
| A0A0H2UHR7 | Filamin-C | Flnc | 0.765902573 | 0.00232869 | … | … | … | … | … | … |
| B2RYG2 | Pck2 protein | Pck2 | 0.829390785 | 0.0341761 | … | … | … | … | … | … |
| B4F7A5 | CD99 molecule (Xg blood group) | Cd99 | 0.683784777 | 0.00391835 | … | … | … | … | … | … |
| B5DEH4 | UDP-N-acetylglucosamine pyrophosphorylase 1-like 1 | Uap1l1 | 1.300415861 | 0.00752714 | … | … | … | … | … | … |
| B5DF34 | Phosphatidylinositol transfer protein, membrane-associated 2 (Predicted), isoform CRA_b | Pitpnm2 | 0.52671088 | 0.00196923 | … | … | … | … | … | … |
| B5DFJ0 | Solute carrier family 7 (Cationic amino acid transporter, y+ system), member 4 | Slc7a4 | 1.369123142 | 0.00180908 | … | … | … | … | … | … |
| B6ID11 | Ubiquitin-related modifier 1 | Urm1 | 1.208464698 | 0.00119353 | … | … | … | … | … | … |
| D3Z937 | High mobility group 20A | Hmg20a | 0.787491465 | 0.0251988 | … | … | … | … | … | … |
| D3ZA89 | Similar to RIKEN cDNA A630047E20 (Predicted) | Tbck | 0.815855283 | 0.00381103 | … | … | … | … | … | … |
| D3ZAQ0 | FUN14 domain-containing 2 | Fundc2 | 0.747373116 | 0.017544 | … | … | … | … | … | … |
| D3ZEI6 | Nuclear receptor coactivator 5 | Ncoa5 | 0.790159045 | 0.0143736 | … | … | … | … | … | … |
| D3ZF11 | Hepatitis B virus x interacting protein (Predicted), isoform CRA_a | Lamtor5 | 0.703878561 | 0.0102479 | … | … | … | … | … | … |
| D3ZI74 | N-acetyltransferase 14 | Nat14 | 0.731888016 | 0.00778321 | … | … | … | … | … | … |
| D3ZL85 | Cytochrome c heme lyase | Hccs | 0.809019307 | 0.0330612 | … | … | … | … | … | … |
| D3ZLA3 | Copine 3 | Cpne3 | 0.665234453 | 0.0134629 | … | … | … | … | … | … |
| D3ZLU0 | C2 calcium-dependent domain-containing 4C | C2cd4c | 0.748404477 | 0.024526 | … | … | … | … | … | … |
| D3ZLX3 | MOB kinase activator 2 | Mob2 | 0.688784977 | 0.00194911 | … | … | … | … | … | … |
| D3ZMR9 | Mitochondrial ribosomal protein L21 | Mrpl21 | 0.697183764 | 0.0426298 | … | … | … | … | … | … |
| D3ZTV7 | ASH2-like histone lysine methyltransferase complex subunit | Ash2l | 1.22677546 | 0.0314638 | … | … | … | … | … | … |
| D3ZU52 | COP9 (Constitutive photomorphogenic) homolog, subunit 7b (Arabidopsis thaliana) (Predicted), isoform CRA_b | Cops7b | 0.729613203 | 0.00148924 | … | … | … | … | … | … |
| D3ZU55 | Forkhead box K1 | Foxk1 | 1.276278329 | 0.00935846 | … | … | … | … | … | … |
| D3ZVN7 | Protoporphyrinogen oxidase | Ppox | 1.293986031 | 0.0129897 | … | … | … | … | … | … |
| D3ZWJ9 | Family with sequence similarity 234, member B | Fam234b | 0.764755814 | 0.0169156 | … | … | … | … | … | … |
| D3ZZA8 | SEC24 homolog A, COPII coat complex component | Sec24a | 0.832964547 | 0.0381307 | … | … | … | … | … | … |
| D3ZZB2 | Peroxisomal biogenesis factor 1 | Pex1 | 0.737318336 | 0.0200729 | … | … | … | … | … | … |
| D3ZZM9 | Centriole, cilia and spindle-associated protein | Ccsap | 0.809467101 | 0.00805997 | … | … | … | … | … | … |
| D3ZZZ5 | Phosphopantothenoylcysteine decarboxylase | Ppcdc | 0.793020392 | 0.00452513 | … | … | … | … | … | … |
| D4A055 | Voltage-dependent L-type calcium channel subunit beta-4 | Cacnb4 | 0.770660467 | 0.000420042 | … | … | … | … | … | … |
| D4A2K5 | Nuclear receptor interacting protein 3 (Predicted), isoform CRA_a | Nrip3 | 0.680296569 | 0.00597096 | … | … | … | … | … | … |
| D4A4H5 | Stromal cell derived factor 2 (Predicted), isoform CRA_b | Sdf2 | 0.697901135 | 0.00420547 | … | … | … | … | … | … |
| D4A4Q4 | OCIA domain-containing 2 | Ociad2 | 1.206346356 | 0.0162339 | … | … | … | … | … | … |
| D4A500 | HD domain containing 3 (Predicted), isoform CRA_b | Hddc3 | 1.258616813 | 0.0149678 | … | … | … | … | … | … |
| D4A5X1 | Stromal interaction molecule 2 | Stim2 | 0.781439434 | 0.0226733 | … | … | … | … | … | … |
| D4A7U6 | U6 snRNA-associated Sm-like protein LSm3 | Lsm3 | 0.685873784 | 0.00322522 | … | … | … | … | … | … |
| D4A831 | Adhesion G protein-coupled receptor B3 | Adgrb3 | 0.806379529 | 0.0317259 | … | … | … | … | … | … |
| D4A8D5 | Filamin B | Flnb | 0.82925536 | 0.0388594 | … | … | … | … | … | … |
| D4A8E7 | COMM domain-containing 6 | Commd6 | 0.767584767 | 0.00833531 | … | … | … | … | … | … |
| D4A9Y0 | RCG36668 | Sdf2l1 | 0.831699244 | 0.00612813 | … | … | … | … | … | … |
| D4AAE5 | Transmembrane protein 56 | Tmem56 | 1.402071633 | 0.0360671 | … | … | … | … | … | … |
| D4ABB8 | Probable phospholipid-transporting ATPase IIB | Atp9b | 1.248677502 | 0.0198962 | … | … | … | … | … | … |
| E9PTV9 | Glyceraldehyde-3-phosphate dehydrogenase | N/A | 0.811174409 | 0.0131767 | … | … | … | … | … | … |
| E9PU17 | ATP-binding cassette sub-family A member 17 | Abca17 | 1.332601329 | 0.0468459 | … | … | … | … | … | … |
| F1LQC1 | Acyl-coenzyme A oxidase | Acox1 | 0.800747175 | 0.0382007 | … | … | … | … | … | … |
| F1LR98 | Cadherin 10 | Cdh10 | 0.794452339 | 0.0445525 | … | … | … | … | … | … |
| F1LS79 | Chondroitin sulfate proteoglycan 4 | Cspg4 | 0.821662714 | 0.0212567 | … | … | … | … | … | … |
| F1LTJ5 | Uncharacterized protein | N/A | 0.791214528 | 0.0149589 | … | … | … | … | … | … |
| F1M0P3 | UAP56-interacting factor | Fyttd1 | 0.805920734 | 0.0184222 | … | … | … | … | … | … |
| G3V6M3 | Synaptotagmin II | Syt2 | 0.765240294 | 0.0427037 | … | … | … | … | … | … |
| G3V709 | Nicotinate phosphoribosyltransferase | Naprt | 0.697487054 | 0.0189095 | … | … | … | … | … | … |
| G3V715 | Non-specific serine/threonine protein kinase | Prkaa2 | 0.780691067 | 0.0172027 | … | … | … | … | … | … |
| G3V7U2 | Microtubule-associated protein 1 A, isoform CRA_c | Map1a | 0.79924397 | 0.0231461 | … | … | … | … | … | … |
| G3V829 | Far upstream element-binding protein 3 | Fubp3 | 0.820852639 | 0.00272028 | … | … | … | … | … | … |
| G8EXB7 | Cytoplasmic polyadenylation element binding protein 2 isoform a | Cpeb2 | 1.225462494 | 0.0101401 | … | … | … | … | … | … |
| H9BFG4 | Interleukin-6 receptor subunit beta | Il6st | 0.795813996 | 0.00278225 | … | … | … | … | … | … |
| M0R4G0 | Immunoglobulin superfamily-containing leucine-rich repeat 2 | Islr2 | 0.740657401 | 0.0381755 | … | … | … | … | … | … |
| M0R608 | Reticulon | Rtn1 | 0.800528933 | 0.0313331 | … | … | … | … | … | … |
| M0R621 | Uncharacterized LOC100909715 | LOC100909715 | 0.78502658 | 0.0201021 | … | … | … | … | … | … |
| M0RD99 | Ribosomal protein L30-like 1 | Rpl30l1 | 0.740916482 | 0.0457276 | … | … | … | … | … | … |
| M0RDK9 | Acyl-CoA dehydrogenase family, member 8 | Acad8 | 0.810026106 | 0.0108267 | … | … | … | … | … | … |
| O08769 | Cyclin dependent kinase inhibitor | Cdkn1b | 0.528947292 | 0.0318822 | … | … | … | … | … | … |
| P01946 | Hemoglobin subunit alpha-1/2 | Hba1 | 0.677777501 | 0.0393905 | … | … | … | … | … | … |
| P02454 | Collagen alpha-1(I) chain | Col1a1 | 1.211307652 | 0.0313441 | … | … | … | … | … | … |
| P0C8E4 | Mitogen-activated protein kinase kinase kinase 7 | Map3k7 | 0.749511651 | 0.039846 | … | … | … | … | … | … |
| P16975 | SPARC | Sparc | 0.734000986 | 0.0117072 | … | … | … | … | … | … |
| P23928 | Alpha-crystallin B chain | Cryab | 0.708855085 | 0.00255785 | … | … | … | … | … | … |
| P37361 | Metallothionein-3 | Mt3 | 1.500198349 | 0.0388621 | … | … | … | … | … | … |
| P40307 | Proteasome subunit beta type-2 | Psmb2 | 1.201151421 | 0.019215 | … | … | … | … | … | … |
| P47861 | Synaptotagmin-5 | Syt5 | 0.701246836 | 0.0388715 | … | … | … | … | … | … |
| P53812 | Phosphatidylinositol transfer protein beta isoform | Pitpnb | 0.80969694 | 0.0265075 | … | … | … | … | … | … |
| P62275 | 40S ribosomal protein S29 | Rps29 | 0.513319875 | 0.00992394 | … | … | … | … | … | … |
| P63025 | Vesicle-associated membrane protein 3 | Vamp3 | 0.718853161 | 0.0212011 | … | … | … | … | … | … |
| P70550 | Ras-related protein Rab-8B | Rab8b | 0.794164415 | 0.00792971 | … | … | … | … | … | … |
| P84039 | Ectonucleotide pyrophosphatase/phosphodiesterase family member 5 | Enpp5 | 1.230965915 | 0.0300514 | … | … | … | … | … | … |
| P97888 | Serine/threonine-protein phosphatase 2A 55 kDa regulatory subunit B gamma isoform | Ppp2r2c | 1.519805775 | 0.0256816 | … | … | … | … | … | … |
| Q0PGW2 | DENN domain-containing protein 11 | Dennd11 | 0.726627693 | 0.019281 | … | … | … | … | … | … |
| Q0VGK2 | Tetratricopeptide repeat protein 39C | Ttc39c | 1.243695871 | 0.0465842 | … | … | … | … | … | … |
| Q3B7K9 | RUN domain-containing protein 3B | Rundc3b | 1.249678426 | 0.0195723 | … | … | … | … | … | … |
| Q3KR80 | Arylsulfatase A | Arsa | 0.816871907 | 0.0207252 | … | … | … | … | … | … |
| Q3SWS9 | Janus kinase and microtubule-interacting protein 1 | Jakmip1 | 0.75558869 | 0.0146643 | … | … | … | … | … | … |
| Q496Z3 | Importin 13, isoform CRA_e | Ipo13 | 0.759750529 | 0.0217871 | … | … | … | … | … | … |
| Q497A9 | Eukaryotic translation initiation factor 4E binding protein 2, isoform CRA_b | Eif4ebp2 | 0.776610487 | 0.0329534 | … | … | … | … | … | … |
| Q498M7 | CCR4-NOT transcription complex, subunit 4 | Cnot4 | 1.278255814 | 0.00124855 | … | … | … | … | … | … |
| Q499S6 | Cathepsin F | Ctsf | 1.257927043 | 0.00670268 | … | … | … | … | … | … |
| Q4QQW3 | Hydroxyacid-oxoacid transhydrogenase, mitochondrial | Adhfe1 | 0.830480736 | 0.0134434 | … | … | … | … | … | … |
| Q4V8C2 | Centromere/kinetochore protein zw10 homolog | Zw10 | 0.735068583 | 0.0332189 | … | … | … | … | … | … |
| Q4V8N0 | Lipocalin 7, isoform CRA_a | Tinagl1 | 0.761637362 | 0.00870383 | … | … | … | … | … | … |
| Q5PPG2 | Legumain | Lgmn | 1.24351734 | 0.0119616 | … | … | … | … | … | … |
| Q5PQL5 | Phosphatidylserine synthase 1 | Ptdss1 | 0.740920567 | 0.0199528 | … | … | … | … | … | … |
| Q5RK24 | Phosphomevalonate kinase | Pmvk | 1.285284863 | 0.00298789 | … | … | … | … | … | … |
| Q5XID7 | Armadillo repeat-containing X-linked protein 3 | Armcx3 | 0.799252117 | 0.000998174 | … | … | … | … | … | … |
| Q5XIF2 | RCG47444, isoform CRA_a | Uros | 0.736366689 | 0.0273573 | … | … | … | … | … | … |
| Q5XIP9 | Transmembrane protein 43 | Tmem43 | 0.826001875 | 0.000563019 | … | … | … | … | … | … |
| Q63666 | Vesicle-associated membrane protein 1 | Vamp1 | 0.826480316 | 0.0117082 | … | … | … | … | … | … |
| Q63707 | Dihydroorotate dehydrogenase (quinone), mitochondrial | Dhodh | 0.7778525 | 0.0393541 | … | … | … | … | … | … |
| Q63910 | Alpha globin | Hba-a3 | 0.368429231 | 0.00474447 | … | … | … | … | … | … |
| Q642A4 | UPF0598 protein C8orf82 homolog | NA | 0.618671253 | 0.032162 | … | … | … | … | … | … |
| Q6AY99 | Aldo-keto reductase family 1, member B10 (Aldose reductase) | Akr1b10 | 1.30240699 | 0.012378 | … | … | … | … | … | … |
| Q6AYS2 | Sideroflexin | Sfxn1 | 0.626150181 | 0.017696 | … | … | … | … | … | … |
| Q6AYS3 | Carboxypeptidase | Ctsa | 0.629632349 | 0.000462962 | … | … | … | … | … | … |
| Q6AZ61 | Probable lysosomal cobalamin transporter | Lmbrd1 | 0.741609543 | 0.00342132 | … | … | … | … | … | … |
| Q6MG51 | Uncharacterized protein C6orf47 homolog | G4 | 0.599534358 | 0.0277319 | … | … | … | … | … | … |
| Q6TEK4 | Vitamin K epoxide reductase complex subunit 1 | Vkorc1 | 0.765546201 | 0.0175083 | … | … | … | … | … | … |
| Q80W89 | NADH dehydrogenase [ubiquinone] 1 alpha subcomplex subunit 11 | Ndufa11 | 0.799984437 | 0.00220729 | … | … | … | … | … | … |
| Q810W7 | Microtubule-associated serine/threonine-protein kinase 1 | Mast1 | 0.803162945 | 0.00347878 | … | … | … | … | … | … |
| Q923M1 | Mitochondrial peptide methionine sulfoxide reductase | Msra | 0.791937715 | 0.00160298 | … | … | … | … | … | … |
| Q99ND9 | RWD domain-containing protein 1 | Rwdd1 | 1.266511443 | 0.0486096 | … | … | … | … | … | … |
| Q9WVK3 | Peroxisomal trans-2-enoyl-CoA reductase | Pecr | 0.735832108 | 0.0286269 | … | … | … | … | … | … |
| Q9Z2Q7 | Syntaxin-8 | Stx8 | 1.209182385 | 0.0151891 | … | … | … | … | … | … |
| A0A0G2JW68 | Synaptotagmin-6 | Syt6 | … | … | … | … | 1.523725511 | 0.0259038 | … | … |
| A0A0G2K1L0 | Tenascin C | Tnc | … | … | … | … | 1.284591591 | 0.016443 | … | … |
| B2RYM5 | Lys-63-specific deubiquitinase BRCC36 | Brcc3 | … | … | … | … | 1.219746678 | 0.0126042 | … | … |
| D0Q0Y7 | Protein cornichon homolog 3 | Cnih3 | … | … | … | … | 0.805950939 | 0.0309869 | … | … |
| D3ZJB8 | RBR-type E3 ubiquitin transferase | Arih2 | … | … | … | … | 0.814231723 | 0.0215042 | … | … |
| D3ZP98 | Histocompatibility 13 (Predicted), isoform CRA_a | Hm13 | … | … | … | … | 0.775949333 | 0.00992381 | … | … |
| D3ZVR7 | Prostamide/prostaglandin F synthase | Prxl2b | … | … | … | … | 1.203598783 | 0.042711 | … | … |
| D4A599 | Dynein, axonemal, heavy chain 17 | Dnah17 | … | … | … | … | 1.274899012 | 0.00492855 | … | … |
| D4AC73 | Coiled-coil-helix-coiled-coil-helix domain-containing 7 | Chchd7 | … | … | … | … | 0.683117266 | 0.00909566 | … | … |
| G3V9J1 | Uncharacterized protein | N/A | … | … | … | … | 1.373440264 | 0.0411794 | … | … |
| P00762 | Anionic trypsin-1 | Prss1 | … | … | … | … | 0.770504785 | 0.037732 | … | … |
| P02770 | Serum albumin | Alb | … | … | … | … | 1.368943554 | 0.00832909 | … | … |
| P05545 | Serine protease inhibitor A3K | Serpina3k | … | … | … | … | 1.336846593 | 0.00993544 | … | … |
| P20760 | Ig gamma-2A chain C region | Igg-2a | … | … | … | … | 1.439418735 | 0.00479689 | … | … |
| P60881 | Synaptosomal-associated protein 25 | Snap25 | … | … | … | … | 0.828858775 | 0.0156896 | … | … |
| Q05175 | Brain acid soluble protein 1 | Basp1 | … | … | … | … | 0.798487608 | 0.0247613 | … | … |
| Q5RKH6 | Protein OS-9 | Os9 | … | … | … | … | 1.351294064 | 0.0400934 | … | … |
| Q66H63 | Ganglioside-induced differentiation-associated-protein 2 | Gdap2 | … | … | … | … | 1.252558397 | 0.00472645 | … | … |
| Q6AZ44 | Acyl-CoA thioesterase 8 | Acot8 | … | … | … | … | 0.761758765 | 0.0494532 | … | … |
| Q7M733 | Hermansky-Pudlak syndrome 6 protein homolog | Hps6 | … | … | … | … | 0.782100955 | 0.0342032 | … | … |
| Q99PD6 | Transforming growth factor beta-1-induced transcript 1 protein | Tgfb1i1 | … | … | … | … | 1.232018648 | 0.0325969 | … | … |
| A0A096MJI4 | Uncharacterized protein | Gigyf2 | … | … | … | … | … | … | 0.585358398 | 0.00829923 |
| A0A096MJJ6 | Acyl-CoA-binding domain-containing protein 5 | Acbd5 | … | … | … | … | … | … | 0.710126017 | 0.0118021 |
| A0A096MJV2 | PDZ domain-containing 11 | Pdzd11 | … | … | … | … | … | … | 0.790602798 | 0.0281767 |
| A0A0A0MXX1 | A-kinase anchor protein 7 isoforms delta and gamma (Fragment) | Akap7 | … | … | … | … | … | … | 1.238503065 | 0.0311488 |
| A0A0G2JSL8 | Adenosine kinase | Adk | … | … | … | … | … | … | 0.820272608 | 0.0139735 |
| A0A0G2JSM7 | Adducin 1 (Alpha), isoform CRA_b | Add1 | … | … | … | … | … | … | 0.762356412 | 0.0314543 |
| A0A0G2JSR0 | Voltage-dependent anion-selective channel protein 3 | Vdac3 | … | … | … | … | … | … | 1.20335513 | 0.0325438 |
| A0A0G2JW13 | NACHT and WD repeat domain-containing protein 2-like | LOC103694210 | … | … | … | … | … | … | 0.831865874 | 0.0260931 |
| A0A0G2JYC7 | Hepatoma-derived growth factor-related protein 2 | Hdgfrp2 | … | … | … | … | … | … | 0.798164257 | 0.0346957 |
| A0A0G2JYV0 | Bromodomain-containing 4 | Brd4 | … | … | … | … | … | … | 0.654982 | 0.0174587 |
| A0A0G2JZY3 | RALBP1-associated Eps domain-containing 1 | Reps1 | … | … | … | … | … | … | 0.785616114 | 0.0326297 |
| A0A0G2K0M6 | 60S ribosomal protein L22 | Rpl22 | … | … | … | … | … | … | 1.203853037 | 0.0268537 |
| A0A0G2K0S0 | Phosphatase and actin regulator | Phactr3 | … | … | … | … | … | … | 0.722637417 | 0.0243124 |
| A0A0G2K0W1 | Disabled homolog 1 | Dab1 | … | … | … | … | … | … | 1.320897583 | 0.0476291 |
| A0A0G2K140 | ERC protein 2 | Erc2 | … | … | … | … | … | … | 0.570530211 | 0.0084426 |
| A0A0G2K285 | Uncharacterized protein | Reps2 | … | … | … | … | … | … | 0.79979047 | 0.0131235 |
| A0A0G2K2L1 | Podocalyxin | Podxl | … | … | … | … | … | … | 0.819621726 | 0.0481224 |
| A0A0G2K2M9 | Serine/arginine repetitive matrix 2 | Srrm2 | … | … | … | … | … | … | 0.771987138 | 0.0181969 |
| A0A0G2K3Y9 | Megakaryoblastic leukemia (Translocation) 1 (Predicted) | Mrtfa | … | … | … | … | … | … | 1.210032074 | 0.015973 |
| A0A0G2K568 | Ketimine reductase mu-crystallin | Crym | … | … | … | … | … | … | 0.816088599 | 0.01098 |
| A0A0G2K5G2 | Cystathionine gamma-lyase | LOC103691744 | … | … | … | … | … | … | 1.246418824 | 0.00310753 |
| A0A0G2K6A2 | Tyrosine-protein phosphatase non-receptor type 9 | Ptpn9 | … | … | … | … | … | … | 0.826862577 | 0.0178237 |
| A0A0G2K8K9 | RNA binding motif protein, X-linked-like 1B | Rbmxl1b | … | … | … | … | … | … | 0.814975587 | 0.00133208 |
| A0A0G2KAJ5 | Coiled-coil domain-containing protein 85A-like | LOC103690141 | … | … | … | … | … | … | 0.811872293 | 0.0266584 |
| A0A0G2KAW7 | Eukaryotic translation initiation factor 4H | Eif4h | … | … | … | … | … | … | 1.218905862 | 0.0435875 |
| A0A0H2UHL8 | Kazrin | Kazn | … | … | … | … | … | … | 0.718127227 | 0.022963 |
| A1L1K3 | Anaphase-promoting complex subunit 5 | Anapc5 | … | … | … | … | … | … | 0.750792038 | 0.00653794 |
| B0BN15 | Similar to src homology 2 domain-containing transforming protein D | Shd | … | … | … | … | … | … | 0.704034155 | 0.0411677 |
| B2GV05 | RNA-binding protein 5 | Rbm5 | … | … | … | … | … | … | 0.802224946 | 0.0141847 |
| B2RYI0 | WD repeat-containing protein 91 | Wdr91 | … | … | … | … | … | … | 0.803723926 | 0.00171297 |
| B2RZD1 | Protein transport protein Sec61 subunit beta | Sec61b | … | … | … | … | … | … | 0.816194823 | 0.0126244 |
| B4F786 | CD2 antigen (Cytoplasmic tail) binding protein 2 (Predicted), isoform CRA_a | Cd2bp2 | … | … | … | … | … | … | 0.802851702 | 0.0117424 |
| B5DFK8 | Pdxdc1 protein | Pdxdc1 | … | … | … | … | … | … | 0.776912041 | 0.0125768 |
| C7EMI4 | Cytochrome b | NA | … | … | … | … | … | … | 1.301478767 | 0.0164905 |
| C9EH87 | Alpha II spectrin | NA | … | … | … | … | … | … | 1.260916235 | 0.0349537 |
| D3Z8Q7 | Cytosolic iron-sulfur assembly component 2B | Ciao2b | … | … | … | … | … | … | 0.731824528 | 0.00722256 |
| D3Z956 | Retinaldehyde binding protein 1 (Predicted), isoform CRA_a | Rlbp1 | … | … | … | … | … | … | 0.776857564 | 7.85E-05 |
| D3Z9C0 | Meiosis regulator for oocyte development | Mios | … | … | … | … | … | … | 0.798103712 | 0.00537669 |
| D3ZAS8 | Spliceosome-associated factor 3, U4/U6 recycling protein | Sart3 | … | … | … | … | … | … | 0.811070915 | 0.0442549 |
| D3ZBL6 | Nucleoporin 160 | Nup160 | … | … | … | … | … | … | 0.665984775 | 0.0469935 |
| D3ZC15 | Capping protein regulator and myosin 1 linker 2 | Carmil2 | … | … | … | … | … | … | 0.797052788 | 0.0131073 |
| D3ZCR3 | High mobility group protein B1 pseudogene | LOC108349189 | … | … | … | … | … | … | 1.204438457 | 0.0046782 |
| D3ZE63 | Uncharacterized protein | N/A | … | … | … | … | … | … | 0.821774236 | 0.0330497 |
| D3ZFY7 | Pleckstrin and Sec7 domain-containing 3 | Psd3 | … | … | … | … | … | … | 1.263843953 | 0.00158807 |
| D3ZIF0 | Zinc finger protein 512 | Zfp512 | … | … | … | … | … | … | 0.800250199 | 0.0200074 |
| D3ZJ92 | Pre-mRNA processing factor 40 homolog A (Yeast) (Predicted) | Prpf40a | … | … | … | … | … | … | 0.755909995 | 0.0207348 |
| D3ZJD3 | Ribosomal_L28e domain-containing protein | RGD1565183 | … | … | … | … | … | … | 1.234445996 | 0.000423859 |
| D3ZJZ1 | Autophagy protein 5 | N/A | … | … | … | … | … | … | 0.746535624 | 0.0377046 |
| D3ZKT0 | Phosphatidate cytidylyltransferase, mitochondrial | Tamm41 | … | … | … | … | … | … | 0.824366654 | 0.0133937 |
| D3ZMK9 | Inactive tyrosine-protein kinase PRAG1 | Prag1 | … | … | … | … | … | … | 0.821584484 | 0.015774 |
| D3ZPT1 | Similar to RIKEN cDNA 1110012L19 | RGD1562747 | … | … | … | … | … | … | 0.702408359 | 0.0279216 |
| D3ZQE8 | Exportin 5 | Xpo5 | … | … | … | … | … | … | 0.827456 | 0.0155861 |
| D3ZQI1 | Glutathione peroxidase | Gpx7 | … | … | … | … | … | … | 0.761147651 | 0.0213621 |
| D3ZS15 | Cytochrome c domain-containing protein | NA | … | … | … | … | … | … | 0.566050583 | 0.000520544 |
| D3ZTX0 | Transmembrane emp24 domain-containing protein 7 | Tmed7 | … | … | … | … | … | … | 1.299489286 | 0.0378919 |
| D3ZXY2 | PDZ domain containing 8 (Predicted) | Pdzd8 | … | … | … | … | … | … | 0.795017518 | 0.0416402 |
| D3ZY71 | RCG21156 | NEWGENE_1586233 | … | … | … | … | … | … | 1.213580114 | 0.0234481 |
| D4A2H8 | Nudix (Nucleoside diphosphate linked moiety X)-type motif 12 (Predicted) | Nudt12 | … | … | … | … | … | … | 1.209164419 | 0.0102293 |
| D4A507 | CAP-Gly domain-containing linker protein 3 | Clip3 | … | … | … | … | … | … | 0.659260195 | 0.00200949 |
| D4A520 | Protein phosphatase | Pptc7 | … | … | … | … | … | … | 0.782470889 | 0.000438924 |
| D4A5P3 | Mitoguardin 1 | Miga1 | … | … | … | … | … | … | 0.793471906 | 0.0248634 |
| D4A6A8 | Protein phosphatase 1, regulatory (Inhibitor) subunit 13B (Predicted) | Ppp1r13b | … | … | … | … | … | … | 0.796495463 | 0.0336248 |
| D4A772 | Dystrobrevin | Dtna | … | … | … | … | … | … | 0.818377656 | 0.0327427 |
| D4A8G0 | LSM12 homolog | Lsm12 | … | … | … | … | … | … | 1.263859354 | 0.0017381 |
| D4AB66 | Stonin-2 | Ston2 | … | … | … | … | … | … | 0.768002168 | 0.04646 |
| D4ACK1 | Nucleoporin 214 | Nup214 | … | … | … | … | … | … | 0.680795759 | 0.00282362 |
| D4ACM9 | Inositol hexakisphosphate and diphosphoinositol-pentakisphosphate kinase 1 | Mfap1a | … | … | … | … | … | … | 1.209463356 | 0.0309927 |
| D4ACU6 | Protein Gpr123 (Fragment) | Adgra1 | … | … | … | … | … | … | 0.647059001 | 0.00124804 |
| E2E1S0 | Cyclin-dependent kinase-like 5 | Cdkl5 | … | … | … | … | … | … | 0.763064584 | 0.0015495 |
| F1LM11 | Pro-neuregulin-2, membrane-bound isoform | Nrg2 | … | … | … | … | … | … | 1.241260658 | 0.046215 |
| F1LN34 | Progressive ankylosis protein homolog | Ankh | … | … | … | … | … | … | 0.811227443 | 0.0453136 |
| F1LN42 | Tensin 1 | Tns1 | … | … | … | … | … | … | 0.688760356 | 0.018843 |
| F1LQ14 | 60S ribosomal protein L34 | Rpl34 | … | … | … | … | … | … | 1.320077541 | 0.00896408 |
| F1LRA4 | Glutamate receptor-interacting protein 1 | Grip1 | … | … | … | … | … | … | 0.822944357 | 0.00444605 |
| F1LV01 | WD repeat domain 19 | Wdr19 | … | … | … | … | … | … | 0.707901602 | 0.00968833 |
| F1LX47 | IQ motif containing with AAA domain | Iqca1 | … | … | … | … | … | … | 1.360578611 | 0.00102765 |
| F1LZJ4 | Putative hydroxypyruvate isomerase | Hyi | … | … | … | … | … | … | 0.734560191 | 0.0366692 |
| F1M0A0 | Anoctamin | Ano3 | … | … | … | … | … | … | 0.785788393 | 0.0183291 |
| F1M4V6 | Kalirin | Kalrn | … | … | … | … | … | … | 0.739773465 | 0.0283265 |
| F1M790 | Prostaglandin F2 receptor negative regulator | Ptgfrn | … | … | … | … | … | … | 0.662579319 | 0.0339547 |
| F1M842 | Tumor protein p53-binding protein 1 | Tp53bp1 | … | … | … | … | … | … | 0.795227689 | 0.00071773 |
| F1M8Y4 | DEP domain-containing MTOR-interacting protein | Deptor | … | … | … | … | … | … | 0.714988918 | 0.00230146 |
| F1M9C0 | Mitogen-activated protein kinase-activated protein kinase 2 | Mapkapk2 | … | … | … | … | … | … | 0.5091148 | 0.0493577 |
| F1M9X4 | Chloride intracellular channel protein | Clic6 | … | … | … | … | … | … | 0.694513126 | 0.0134504 |
| F7F3Z1 | Lectin, mannose-binding 2-like | Lman2l | … | … | … | … | … | … | 0.795127682 | 0.00822319 |
| G3V6D9 | Na(+)/H(+) exchange regulatory cofactor NHE-RF | Slc9a3r2 | … | … | … | … | … | … | 0.79439737 | 0.0160397 |
| G3V6X7 | ProSAAS | LOC108348172 | … | … | … | … | … | … | 0.81576001 | 0.0430999 |
| G3V6Z5 | Glutamate receptor 3 | Gria3 | … | … | … | … | … | … | 1.299081565 | 0.00332792 |
| G3V7Z6 | ATP-binding cassette sub-family D member 2 | Abcd2 | … | … | … | … | … | … | 1.220560052 | 0.0188775 |
| I7EFB0 | Myelin basic protein transcript variant N (Fragment) | Mbp | … | … | … | … | … | … | 1.406776589 | 0.0154841 |
| L0N559 | Anion exchange protein | Slc4a5 | … | … | … | … | … | … | 1.229860747 | 0.0456562 |
| M0R6K5 | Uncharacterized protein (Fragment) | Adgrv1 | … | … | … | … | … | … | 0.786604778 | 0.0169795 |
| M0R749 | Tetraspanin | Tspan15 | … | … | … | … | … | … | 1.227744032 | 0.0160289 |
| M0RAD5 | ATP-dependent Clp protease proteolytic subunit | Clpp | … | … | … | … | … | … | 1.208348989 | 0.0479154 |
| M0RCH4 | Gp_dh_C domain-containing protein | NA | … | … | … | … | … | … | 0.821073482 | 0.0451234 |
| O08871 | G-protein coupled inward rectifier potassium channel splice variant Kir3.1b (Fragment) | NA | … | … | … | … | … | … | 1.234203192 | 0.0151337 |
| O35119 | Short transient receptor potential channel 4 | Trpc4 | … | … | … | … | … | … | 0.814755492 | 0.0382408 |
| O35147 | Bcl2-associated agonist of cell death | Bad | … | … | … | … | … | … | 0.819609538 | 0.0186545 |
| O35276 | Neuropilin-2 | Nrp2 | … | … | … | … | … | … | 0.803305611 | 0.00427248 |
| O55215 | Ribosomal protein S2 | N/A | … | … | … | … | … | … | 1.203990987 | 0.00214515 |
| O88267 | Acyl-coenzyme A thioesterase 1 | Acot1 | … | … | … | … | … | … | 0.732215265 | 0.00479835 |
| O88370 | Phosphatidylinositol 5-phosphate 4-kinase type-2 gamma | Pip4k2c | … | … | … | … | … | … | 1.203412241 | 0.0161565 |
| P01041 | Cystatin-B | Cstb | … | … | … | … | … | … | 1.267697781 | 0.0467274 |
| P02696 | Retinol-binding protein 1 | Rbp1 | … | … | … | … | … | … | 0.808180936 | 0.0114757 |
| P04762 | Catalase | Cat | … | … | … | … | … | … | 0.828349028 | 0.0150265 |
| P05714 | Ras-related protein Rab-4A | Rab4a | … | … | … | … | … | … | 1.203011352 | 0.0140073 |
| P0C2C0 | 39S ribosomal protein L22, mitochondrial | Mrpl22 | … | … | … | … | … | … | 0.735370993 | 0.00279634 |
| P0C627 | UBX domain-containing protein 2B | Ubxn2b | … | … | … | … | … | … | 0.821692148 | 0.0326802 |
| P10818 | Cytochrome c oxidase subunit 6A1, mitochondrial | Cox6a1 | … | … | … | … | … | … | 1.214591005 | 0.0352703 |
| P13668 | Stathmin | Stmn1 | … | … | … | … | … | … | 1.225058049 | 0.0296223 |
| P14841 | Cystatin-C | Cst3 | … | … | … | … | … | … | 1.228949354 | 0.0343356 |
| P18508 | Gamma-aminobutyric acid receptor subunit gamma-2 | Gabrg2 | … | … | … | … | … | … | 0.704559009 | 0.0138141 |
| P23565 | Alpha-internexin | Ina | … | … | … | … | … | … | 1.314286234 | 0.000845217 |
| P25122 | Potassium voltage-gated channel subfamily C member 1 | Kcnc1 | … | … | … | … | … | … | 0.807536784 | 0.0318814 |
| P25886 | 60S ribosomal protein L29 | Rpl29 | … | … | … | … | … | … | 1.450820261 | 0.0126959 |
| P26431 | Sodium/hydrogen exchanger 1 | Slc9a1 | … | … | … | … | … | … | 0.830519409 | 0.00276404 |
| P36860 | Ras-related protein Ral-B | Ralb | … | … | … | … | … | … | 1.267179513 | 0.0178581 |
| P41413 | Proprotein convertase subtilisin/kexin type 5 | Pcsk5 | … | … | … | … | … | … | 1.203246123 | 0.0222281 |
| P47875 | Cysteine and glycine-rich protein 1 | Csrp1 | … | … | … | … | … | … | 1.210723583 | 0.0285843 |
| P52555 | Endoplasmic reticulum resident protein 29 | Erp29 | … | … | … | … | … | … | 0.807236064 | 0.0114284 |
| P61354 | 60S ribosomal protein L27 | Rpl27 | … | … | … | … | … | … | 1.206215881 | 0.0141076 |
| P62255 | Ubiquitin-conjugating enzyme E2 G1 | Ube2g1 | … | … | … | … | … | … | 0.779463582 | 0.0147178 |
| P62845 | 40S ribosomal protein S15 | Rps15 | … | … | … | … | … | … | 1.259471061 | 0.00192819 |
| P63081 | V-type proton ATPase 16 kDa proteolipid subunit | Atp6v0c | … | … | … | … | … | … | 0.817817457 | 0.0220957 |
| P67999 | Ribosomal protein S6 kinase beta-1 | Rps6kb1 | … | … | … | … | … | … | 0.774417812 | 0.00216706 |
| P81795 | Eukaryotic translation initiation factor 2 subunit 3, X-linked | Eif2s3 | … | … | … | … | … | … | 0.735317349 | 0.0424247 |
| Q08326 | Guanine nucleotide exchange factor MSS4 | Rabif | … | … | … | … | … | … | 0.795536573 | 0.0391421 |
| Q0VGK4 | Lysophospholipase D GDPD1 | Gdpd1 | … | … | … | … | … | … | 1.217959764 | 0.00893113 |
| Q32PX2 | Aminoacyl tRNA synthase complex-interacting multifunctional protein 2 | Aimp2 | … | … | … | … | … | … | 0.801113964 | 0.0467212 |
| Q3T1K9 | Nicolin 1 | Nicn1 | … | … | … | … | … | … | 0.709892436 | 0.0115912 |
| Q498T9 | Volume-regulated anion channel subunit LRRC8C | Lrrc8c | … | … | … | … | … | … | 0.83324805 | 0.0198528 |
| Q4KM31 | LIM domain-containing protein 2 | Limd2 | … | … | … | … | … | … | 1.462111842 | 0.00626668 |
| Q4QQT5 | Probable isocitrate dehydrogenase [NAD] gamma 2, mitochondrial | N/A | … | … | … | … | … | … | 0.810673207 | 0.018611 |
| Q4V8H8 | EH domain-containing protein 2 | Ehd2 | … | … | … | … | … | … | 0.796732833 | 0.00958429 |
| Q562C9 | 1,2-dihydroxy-3-keto-5-methylthiopentene dioxygenase | Adi1 | … | … | … | … | … | … | 0.73449832 | 0.00136247 |
| Q5BJS9 | MORN repeat-containing protein 4 | Morn4 | … | … | … | … | … | … | 0.771378461 | 0.0133986 |
| Q5FVQ8 | NLR family member X1 | Nlrx1 | … | … | … | … | … | … | 0.738160761 | 0.0112806 |
| Q5PPJ4 | Deoxyhypusine hydroxylase | Dohh | … | … | … | … | … | … | 0.811976027 | 0.0035456 |
| Q5PPN5 | Tubulin polymerization-promoting protein family member 3 | Tppp3 | … | … | … | … | … | … | 1.201956625 | 0.00422116 |
| Q5PQJ6 | Pyrroline-5-carboxylate reductase 3 | Pycr3 | … | … | … | … | … | … | 0.830330496 | 0.0125365 |
| Q5PQN9 | 39S ribosomal protein L38, mitochondrial | Mrpl38 | … | … | … | … | … | … | 0.780991745 | 0.0261131 |
| Q5U2R3 | FERM domain-containing protein 8 | Frmd8 | … | … | … | … | … | … | 0.71950816 | 0.0269809 |
| Q5U3Z0 | Cilia- and flagella-associated protein 298 | Cfap298 | … | … | … | … | … | … | 0.794211763 | 0.0444433 |
| Q5XI79 | Protein arginine methyltransferase NDUFAF7, mitochondrial | Ndufaf7 | … | … | … | … | … | … | 0.782332192 | 0.0121863 |
| Q5XID0 | Protein YIPF5 | Yipf5 | … | … | … | … | … | … | 0.663615867 | 0.00331726 |
| Q62806 | Zinc finger protein 148 | Znf148 | … | … | … | … | … | … | 0.790485722 | 0.0188946 |
| Q63016 | Large neutral amino acids transporter small subunit 1 | Slc7a5 | … | … | … | … | … | … | 0.819070616 | 0.0308316 |
| Q63259 | Receptor-type tyrosine-protein phosphatase-like N | Ptprn | … | … | … | … | … | … | 0.809756149 | 0.0452503 |
| Q63524 | Transmembrane emp24 domain-containing protein 2 | Tmed2 | … | … | … | … | … | … | 1.330145037 | 0.0399831 |
| Q63788 | Phosphatidylinositol 3-kinase regulatory subunit beta | Pik3r2 | … | … | … | … | … | … | 0.735873261 | 0.0415235 |
| Q641X9 | 39S ribosomal protein L9, mitochondrial | Mrpl9 | … | … | … | … | … | … | 0.742934097 | 0.0042547 |
| Q64259 | Von Hippel-Lindau disease tumor suppressor | Vhl | … | … | … | … | … | … | 0.707061696 | 0.0248403 |
| Q66H91 | G protein-coupled receptor kinase interacting ArfGAP 2 | Git2 | … | … | … | … | … | … | 0.672069369 | 0.0262387 |
| Q66HG8 | Protein Red | Ik | … | … | … | … | … | … | 0.698243924 | 0.0331341 |
| Q68FY1 | Nucleoporin NUP53 | Nup35 | … | … | … | … | … | … | 0.706716358 | 0.0178861 |
| Q68G39 | Cyclin-dependent kinase 16 | Cdk16 | … | … | … | … | … | … | 0.775033549 | 0.0476034 |
| Q6AYA4 | Polypeptide N-acetylgalactosaminyltransferase | Galnt14 | … | … | … | … | … | … | 0.637492114 | 0.0389339 |
| Q6IN37 | GM2 ganglioside activator | Gm2a | … | … | … | … | … | … | 0.776596531 | 0.029678 |
| Q6MGA9 | Bromodomain-containing protein 2 | Brd2 | … | … | … | … | … | … | 0.804401746 | 0.00659334 |
| Q6P7S0 | Pyruvate kinase | N/A | … | … | … | … | … | … | 1.247065616 | 0.0414874 |
| Q6PCT8 | Succinate dehydrogenase [ubiquinone] cytochrome b small subunit, mitochondrial | Sdhd | … | … | … | … | … | … | 1.216238494 | 0.00576814 |
| Q6RUV5 | Ras-related C3 botulinum toxin substrate 1 | Rac1 | … | … | … | … | … | … | 1.209041752 | 0.0310676 |
| Q765A7 | GPI inositol-deacylase | Pgap1 | … | … | … | … | … | … | 1.265447664 | 0.0407037 |
| Q7M0B5 | Protein-tyrosine kinase IIB, 38K | N/A | … | … | … | … | … | … | 1.235832776 | 0.0245227 |
| Q7TP42 | Ab2-292 | Sec62 | … | … | … | … | … | … | 1.206849323 | 0.00503826 |
| Q8CFC9 | Leucine zipper putative tumor suppressor 1 | Lzts1 | … | … | … | … | … | … | 0.7950435 | 0.00304287 |
| Q8VIF7 | Methanethiol oxidase | Selenbp1 | … | … | … | … | … | … | 0.831147305 | 0.0207991 |
| Q920H8 | Hephaestin | Heph | … | … | … | … | … | … | 0.815344173 | 0.0082204 |
| Q924Y3 | Claudin | N/A | … | … | … | … | … | … | 1.288298655 | 0.00563031 |
| Q9JHB5 | Translin-associated protein X | Tsnax | … | … | … | … | … | … | 0.815670223 | 0.0323872 |
| Q9JHM3 | MHC class I antigen | RT1 | … | … | … | … | … | … | 0.703888762 | 0.0343123 |
| Q9JHY2 | Sideroflexin-3 | Sfxn3 | … | … | … | … | … | … | 1.520047737 | 0.0154815 |
| Q9JJW1 | Tetraspanin-2 | Tspan2 | … | … | … | … | … | … | 1.282600255 | 0.0383911 |
| Q9JKM5 | Sphingosine 1-phosphate receptor 5 | S1pr5 | … | … | … | … | … | … | 0.689618825 | 0.0224114 |
| Q9WU61 | Chloride channel CLIC-like protein 1 | Clcc1 | … | … | … | … | … | … | 0.795394907 | 0.00888755 |
| Q9Z2T2 | Potassium channel subfamily K member 1 | Kcnk1 | … | … | … | … | … | … | 0.817785057 | 0.0205542 |
| A0A0G2JZZ4 | Selenoprotein M | Selenom | … | … | 1.207492587 | 0.040636 | … | … | … | … |
| A0A0G2K3P9 | Synaptogyrin-1 | Syngr1 | … | … | 1.242938229 | 0.0364028 | … | … | … | … |
| A0A0G2K7U7 | Ankyrin repeat domain 27 | Ankrd27 | … | … | 1.51681602 | 0.046667 | … | … | … | … |
| A0A0G2K857 | Kinesin-like protein | Kifc2 | … | … | 1.302203016 | 0.0139607 | … | … | … | … |
| A0JN25 | Microtubule-associated protein | Mapt | … | … | 0.826493732 | 0.0175545 | … | … | … | … |
| A0PJ39 | Voltage-dependent L-type calcium channel subunit alpha | Cacna1c | … | … | 1.40372522 | 6.04E-05 | … | … | … | … |
| B1WC38 | Xpr1 protein | Xpr1 | … | … | 0.786255331 | 0.0228283 | … | … | … | … |
| B2GVB4 | Sept9 protein | Sept9 | … | … | 1.299510386 | 0.0134941 | … | … | … | … |
| B2RYB6 | Aars2 protein | Aars2 | … | … | 1.210234533 | 0.0429153 | … | … | … | … |
| B2RYF7 | WASH complex subunit 1 | Washc1 | … | … | 1.206980921 | 0.0300525 | … | … | … | … |
| D3ZBP2 | SIN3 transcription regulator family member A | Sin3a | … | … | 0.753931322 | 0.021044 | … | … | … | … |
| D3ZKL3 | Cytoplasmic polyadenylation element binding protein 4 (Predicted) | Cpeb4 | … | … | 1.238785851 | 0.0298023 | … | … | … | … |
| D4A5D4 | Microtubule crosslinking factor 1 | Mtcl1 | … | … | 0.767882731 | 0.029392 | … | … | … | … |
| D4A6E8 | Similar to RIKEN cDNA 2410004B18 | RGD1560065 | … | … | 1.257537738 | 0.0398367 | … | … | … | … |
| D4A6V3 | dUTPase domain-containing protein | N/A | … | … | 1.32261073 | 0.0276271 | … | … | … | … |
| D4A7X5 | Protein phosphatase 1K (PP2C domain containing) (Predicted) | Ppm1k | … | … | 1.324838073 | 0.00711669 | … | … | … | … |
| D4ABX8 | Leucine-rich repeat and fibronectin type-III domain-containing protein 4 | Lrfn4 | … | … | 1.299910257 | 0.00317969 | … | … | … | … |
| F1M1C9 | Phosphorylase b kinase regulatory subunit | N/A | … | … | 1.261864683 | 0.0385139 | … | … | … | … |
| F1M8F4 | Glia maturation factor | Gmfg | … | … | 0.743376213 | 0.00448751 | … | … | … | … |
| F1M9F9 | Jouberin | Ahi1 | … | … | 1.2110534 | 0.00324031 | … | … | … | … |
| G3V8M5 | Serine/threonine-protein phosphatase | Ppp4c | … | … | 1.257133022 | 0.0364845 | … | … | … | … |
| G3V963 | RCG47487, isoform CRA_b | Tmem132a | … | … | 1.231561883 | 0.0422186 | … | … | … | … |
| M0RCJ9 | Protein MAL2-like | LOC100911027 | … | … | 1.213093063 | 0.0497841 | … | … | … | … |
| P02688 | Myelin basic protein | Mbp | … | … | 0.729515412 | 0.0413664 | … | … | … | … |
| P04218 | OX-2 membrane glycoprotein | Cd200 | … | … | 1.366759273 | 0.0283437 | … | … | … | … |
| P31232 | Transgelin | Tagln | … | … | 0.791350791 | 0.010809 | … | … | … | … |
| Q04940 | Neurogranin | Nrgn | … | … | 0.826222936 | 0.0181052 | … | … | … | … |
| Q05140 | Clathrin coat assembly protein AP180 | Snap91 | … | … | 0.815343077 | 0.035544 | … | … | … | … |
| Q2KMM2 | Trafficking protein particle complex subunit 1 | Trappc1 | … | … | 0.755479655 | 0.037419 | … | … | … | … |
| Q3KR85 | Phosphoglycerate dehydrogenase like 1 | Ubac2 | … | … | 1.320366031 | 0.00453693 | … | … | … | … |
| Q505J5 | Synaptotagmin XI | Syt11 | … | … | 1.290144886 | 0.0251179 | … | … | … | … |
| Q5BJU5 | Protein cornichon homolog 2 | Cnih2 | … | … | 1.207071329 | 0.0494753 | … | … | … | … |
| Q5FVG5 | Similar to tropomyosin 1, embryonic fibroblast-rat, isoform CRA_c | Tpm2 | … | … | 0.787862901 | 0.0178815 | … | … | … | … |
| Q5M7W5 | Microtubule-associated protein 4 | Map4 | … | … | 0.768299111 | 0.0136227 | … | … | … | … |
| Q5NSW3 | Myelin protein zero | P0 | … | … | 0.722696393 | 0.0131146 | … | … | … | … |
| Q5XJW2 | Growth arrest and DNA damage-inducible proteins-interacting protein 1 | Gadd45gip1 | … | … | 1.202456764 | 0.01358 | … | … | … | … |
| Q64644 | Choline dehydrogenase | RRCHDH | … | … | 1.202047234 | 0.0444652 | … | … | … | … |
| Q6AYR2 | Protein NDRG3 | Ndrg3 | … | … | 1.247243692 | 0.0339523 | … | … | … | … |
| Q6PEC2 | Mrpl16 protein | Mrpl16 | … | … | 1.206617627 | 0.0335123 | … | … | … | … |
| Q6Q0N0 | Calsyntenin-1 | Clstn1 | … | … | 0.827367165 | 0.0274334 | … | … | … | … |
| Q920M2 | RING finger protein 39 | Rnf39 | … | … | 1.26044313 | 0.019202 | … | … | … | … |

**Supplementary Table S3:** The detailed information of GO analysis of 529 differentially expressed proteins in the four stress-induced depression models.

| **Category** | **GO-Term** | **Count** | **PValue** |
| --- | --- | --- | --- |
| Biological processes | vesicle fusion | 8 | 8.56E-05 |
| Biological processes | calcium ion-regulated exocytosis of neurotransmitter | 7 | 1.25E-04 |
| Biological processes | response to hydrogen peroxide | 9 | 2.19E-04 |
| Biological processes | protein transport | 16 | 3.44E-04 |
| Biological processes | oxygen transport | 5 | 4.53E-04 |
| Biological processes | synaptic transmission, GABAergic | 4 | 0.002766627 |
| Biological processes | proteolysis involved in cellular protein catabolic process | 6 | 0.004121406 |
| Biological processes | regulation of calcium ion-dependent exocytosis | 5 | 0.004736494 |
| Biological processes | neurotransmitter secretion | 5 | 0.005866111 |
| Biological processes | response to nutrient | 9 | 0.008116671 |
| Biological processes | growth hormone secretion | 3 | 0.008512297 |
| Biological processes | exocytosis | 7 | 0.010250306 |
| Biological processes | L-methionine biosynthetic process from methylthioadenosine | 3 | 0.011193996 |
| Biological processes | regulation of autophagy | 5 | 0.014190356 |
| Biological processes | protein secretion | 4 | 0.01475911 |
| Biological processes | protein heterooligomerization | 7 | 0.018352279 |
| Biological processes | negative regulation of TOR signaling | 4 | 0.020080807 |
| Biological processes | negative regulation of reactive oxygen species metabolic process | 4 | 0.020080807 |
| Biological processes | synaptic transmission, glutamatergic | 4 | 0.022059169 |
| Biological processes | response to glucose | 7 | 0.02370528 |
| Biological processes | positive regulation of neuron death | 4 | 0.024140015 |
| Biological processes | response to hypoxia | 12 | 0.026797016 |
| Biological processes | translation | 15 | 0.028089863 |
| Biological processes | response to amino acid | 5 | 0.032780235 |
| Biological processes | neuron projection development | 8 | 0.033684263 |
| Biological processes | negative regulation of insulin receptor signaling pathway | 4 | 0.03876281 |
| Biological processes | cellular response to starvation | 5 | 0.040432017 |
| Biological processes | neurological system process | 3 | 0.04299214 |
| Biological processes | transport | 8 | 0.044218195 |
| Biological processes | neuron migration | 7 | 0.045715053 |
| Cellular components | postsynaptic density | 23 | 5.13E-09 |
| Cellular components | cell junction | 30 | 9.21E-09 |
| Cellular components | synapse | 21 | 3.53E-06 |
| Cellular components | mitochondrion | 60 | 9.46E-06 |
| Cellular components | peroxisomal membrane | 9 | 1.06E-05 |
| Cellular components | focal adhesion | 23 | 2.87E-05 |
| Cellular components | cytosol | 58 | 2.95E-05 |
| Cellular components | myelin sheath | 15 | 4.57E-05 |
| Cellular components | membrane | 72 | 8.67E-05 |
| Cellular components | endoplasmic reticulum membrane | 27 | 1.01E-04 |
| Cellular components | hemoglobin complex | 5 | 2.05E-04 |
| Cellular components | endoplasmic reticulum | 34 | 2.19E-04 |
| Cellular components | lysosome | 15 | 5.87E-04 |
| Cellular components | cell projection | 9 | 8.46E-04 |
| Cellular components | terminal bouton | 10 | 8.76E-04 |
| Cellular components | peroxisome | 10 | 9.85E-04 |
| Cellular components | synaptic vesicle | 10 | 0.00145975 |
| Cellular components | dendritic shaft | 7 | 0.001468973 |
| Cellular components | axon | 18 | 0.001478649 |
| Cellular components | neuron projection | 18 | 0.003512419 |
| Cellular components | ribosome | 10 | 0.003881167 |
| Cellular components | perinuclear region of cytoplasm | 25 | 0.003986625 |
| Cellular components | cytoplasm | 133 | 0.004130997 |
| Cellular components | presynaptic membrane | 7 | 0.004271415 |
| Cellular components | recycling endosome | 8 | 0.006816819 |
| Cellular components | recycling endosome membrane | 5 | 0.006939753 |
| Cellular components | synaptic vesicle membrane | 6 | 0.007394568 |
| Cellular components | postsynaptic membrane | 11 | 0.008078906 |
| Cellular components | extracellular exosome | 73 | 0.008231975 |
| Cellular components | cell surface | 23 | 0.008829395 |
| Cellular components | chloride channel complex | 5 | 0.011773405 |
| Cellular components | dendritic spine | 9 | 0.011974747 |
| Cellular components | mitochondrial outer membrane | 8 | 0.012408995 |
| Cellular components | Golgi apparatus | 28 | 0.013273113 |
| Cellular components | cell body | 7 | 0.015383973 |
| Cellular components | intracellular membrane-bounded organelle | 24 | 0.01670728 |
| Cellular components | vesicle | 9 | 0.018998442 |
| Cellular components | AMPA glutamate receptor complex | 4 | 0.023574167 |
| Cellular components | cytoskeleton | 11 | 0.02888358 |
| Cellular components | protein complex | 21 | 0.029722185 |
| Cellular components | neuronal cell body | 19 | 0.032733503 |
| Cellular components | endoplasmic reticulum-Golgi intermediate compartment | 5 | 0.03736254 |
| Cellular components | trans-Golgi network | 8 | 0.04247099 |
| Cellular components | dendrite | 17 | 0.042919506 |
| Cellular components | mitochondrial large ribosomal subunit | 4 | 0.046364267 |
| Molecular function | protein binding | 67 | 5.02E-08 |
| Molecular function | SNARE binding | 8 | 2.81E-04 |
| Molecular function | oxygen binding | 6 | 3.69E-04 |
| Molecular function | oxygen transporter activity | 5 | 4.65E-04 |
| Molecular function | poly(A) RNA binding | 41 | 9.01E-04 |
| Molecular function | syntaxin binding | 8 | 0.001934533 |
| Molecular function | protein serine/threonine phosphatase activity | 6 | 0.003892767 |
| Molecular function | calcium-dependent phospholipid binding | 6 | 0.004615153 |
| Molecular function | clathrin binding | 6 | 0.005009933 |
| Molecular function | protein transporter activity | 6 | 0.014964521 |
| Molecular function | structural constituent of myelin sheath | 3 | 0.017735502 |
| Molecular function | chloride channel activity | 5 | 0.022096399 |
| Molecular function | translation factor activity, RNA binding | 3 | 0.025302717 |
| Molecular function | microtubule binding | 10 | 0.03413731 |
| Molecular function | ATPase binding | 6 | 0.036389639 |
| Molecular function | protein phosphatase binding | 6 | 0.039539553 |
| Molecular function | inositol-1,3,4,5,6-pentakisphosphate kinase activity | 2 | 0.041601395 |
| Molecular function | SNAP receptor activity | 4 | 0.04228018 |

GO analysis of differentially expressed proteins in the four models identified GO terms for 30 important biological processes (BP), 45 cellular components (CC) and 18 molecular functions (MF).
